# An automated growth enclosure for metabolic labeling of *Arabidopsis thaliana *with ^13^C-carbon dioxide - an *in vivo *labeling system for proteomics and metabolomics research

**DOI:** 10.1186/1477-5956-9-9

**Published:** 2011-02-10

**Authors:** Wen-Ping Chen, Xiao-Yuan Yang, Geoffrey L Harms, William M Gray, Adrian D Hegeman, Jerry D Cohen

**Affiliations:** 1 Department of Horticultural Science, University of Minnesota, Saint Paul, USA; 2 Department of Plant Biology, University of Minnesota, Saint Paul, USA; 3 Microbial and Plant Genomics Institute, University of Minnesota, Saint Paul, USA; 4 Saint Paul Apparatus Shop, University of Minnesota, Saint Paul, USA; 5 Yeastern Biotech Co., Ltd. 6F, 23, Lane 169, Kang Ning St., Shijr, Taipei, Taiwan; 6 Room S1-411, Institute of Genetics and Developmental Biology, Chinese Academy of Sciences, No. 1 West BeiChen road, ChaoYang district, Beijing, PR China

## Abstract

**Background:**

Labeling whole *Arabidopsis (Arabidopsis thaliana) *plants to high enrichment with ^13^C for proteomics and metabolomics applications would facilitate experimental approaches not possible by conventional methods. Such a system would use the plant's native capacity for carbon fixation to ubiquitously incorporate ^13^C from ^13^CO_2 _gas. Because of the high cost of ^13^CO_2 _it is critical that the design conserve the labeled gas.

**Results:**

A fully enclosed automated plant growth enclosure has been designed and assembled where the system simultaneously monitors humidity, temperature, pressure and ^13^CO_2 _concentration with continuous adjustment of humidity, pressure and ^13^CO_2 _levels controlled by a computer running LabView software. The enclosure is mounted on a movable cart for mobility among growth environments. *Arabidopsis *was grown in the enclosure for up to 8 weeks and obtained on average >95 atom% enrichment for small metabolites, such as amino acids and >91 atom% for large metabolites, including proteins and peptides.

**Conclusion:**

The capability of this labeling system for isotope dilution experiments was demonstrated by evaluation of amino acid turnover using GC-MS as well as protein turnover using LC-MS/MS. Because this 'open source' *Arabidopsis *^13^C-labeling growth environment was built using readily available materials and software, it can be adapted easily to accommodate many different experimental designs.

## Background

Radioactive and stable isotope tracing techniques have been used for decades and have yielded revolutionary insights into plant metabolism, including photorespiration [1-3] and photosynthetic carbon assimilation [4-6]. These techniques have also been utilized to study secondary plant metabolites [2,7,8] and to understand carbon flux from plants to soil organisms [9] or within plants in different seasons [10] or under stress [11]. Similarly, the pioneering work of Rittenberg and Foster [12] on stable isotope dilution analysis revolutionized the ability for quantitative analysis of low abundance labile compounds and quantitative analysis of such compounds *in vivo *[13-15]. Stable isotope dilution has become the *de facto *standard for analysis of phytohormones and related compounds in plant tissues [16,17].

In the post-genomic era of biological research, there has been increasing interest in making the connections between gene expression and the mechanisms of metabolic regulation in response to internal stimulation and external perturbation. Because carbon atoms are present in virtually all metabolites and cellular macromolecules, labeling plants with [^13^C]-labeled tracers, such as ^13^CO_2 _[18,19] and [^13^C]glucose [20-23] has been used to monitor fluxes of metabolites in isotopologue perturbation/relaxation experiments as well as to generate universal and highly enriched internal standards for metabolite profiling in whole biological systems [24,25]. Using [^13^C]-labeled metabolites extracted from labeled plants for the quantification of multiple metabolites in plants has significant advantages over using single internal standards despite the fact that single compound standards have been routinely used for metabolite profiling [26-29]. Recent labeling studies with [^13^C]glucose and ^13^CO_2 _suggest that the ^13^C isotope effects are of insufficient magnitude to detectably perturb the fluxes and enzymatic kinetics of the metabolic networks within the error found in typical analyses [19,22]. Flux analysis using [^13^C]-labeled tracers needs to be performed with care; however, as the omission of reaction pathways or a failure to account for metabolite channeling can result in significant errors [30].

Plants can be easily labeled with ^13^CO_2 _or ^14^CO_2 _because the tracers can be readily assimilated into the metabolic systems either via ribulose-1,5-bisphosphate carboxylase oxygenase (Rubisco; EC 4.1.1.39) or phosphoenolpyruvate carboxylase (EC 4.1.1.31). Various methods for partial or ubiquitous ^13^C labeling have previously been reported for plants using different labeling systems, as summarized below. ^13^CO_2 _tracer experiments have been conducted by short exposure (ranging from few minutes to few days) of plant leaves to a ^13^CO_2 _environment using simple leaf clamps or cuvettes [2,31], a plastic bag [10], or delicate leaf chambers [1,3,7,32] with or without humidity control. Labeling has also been accomplished using a simple sealed flask containing ^13^CO_2 _for plant tissue cultures [6]. Air-tight enclosures with basic humidity regulation have been designed to partially label whole plants with ^13^CO_2 _for metabolite flux studies [8,9,33]. Pulse labeling studies with ^13^CO_2 _usually resulted in low [^13^C]-enrichments of the metabolites of interest. Even though the final [^13^C]-enrichment may not directly influence the accuracy of flux determination, there is no doubt that increasing labeling homogeneity and ^13^C enrichment for all metabolite pools will lead to higher experimental reproducibility for isotope dilution studies. Recently, a commercially available enclosure, called BioBox, was shown to label whole plants with ^13^CO_2_. Plants from BioBox experiments were subsequently used for flux studies [18,19] and to generate highly labeled metabolites to be used as internal standards for metabolite profiling [25]. However, as the BioBox is a proprietary commercial product, there is minimal information available in the public domain regarding specifications, components or functional characteristics.

In this study, we describe an automated growth enclosure for the [^13^C]-labeling of whole *Arabidopsis *plants. In contrast to the commercial system, this 'open source' enclosure was constructed using components that are either readily available from suppliers or can be easily manufactured from available materials. Additionally, the control software was implemented using the readily accessible and easily modified LabView environment (National Instruments, Austin, TX, USA) with the code included in Additional file 1. An expandable modular enclosure design was used to provide flexibility for optimal use of space and to maximize ^13^CO_2 _use efficiency. The enclosure is portable and is designed for placement within a larger environmental growth chamber to allow for ease of control of external light quantity as well as quality and to allow better temperature control than is possible with internal enclosure lighting systems.

Highly enriched metabolites, such as amino acids, as well as larger molecules, such as proteins, were obtained from *Arabidopsis *after a 3-week labeling period with 99atom% ^13^CO_2_. We also demonstrated that the plants generated by this enclosed system could be used to study amino acid and protein turnover in isotopic dilution experiments after a ^13^CO_2 _labeling period. Our results indicate that the ^13^CO_2 _enclosure is suitable for the analysis of metabolic and protein flux and for the generation of highly enriched plant metabolites usable as universal internal standards for metabolic and protein profiling research.

## Results

### Construction of the ^13^CO_2 _labeling system

**Figure 1:**
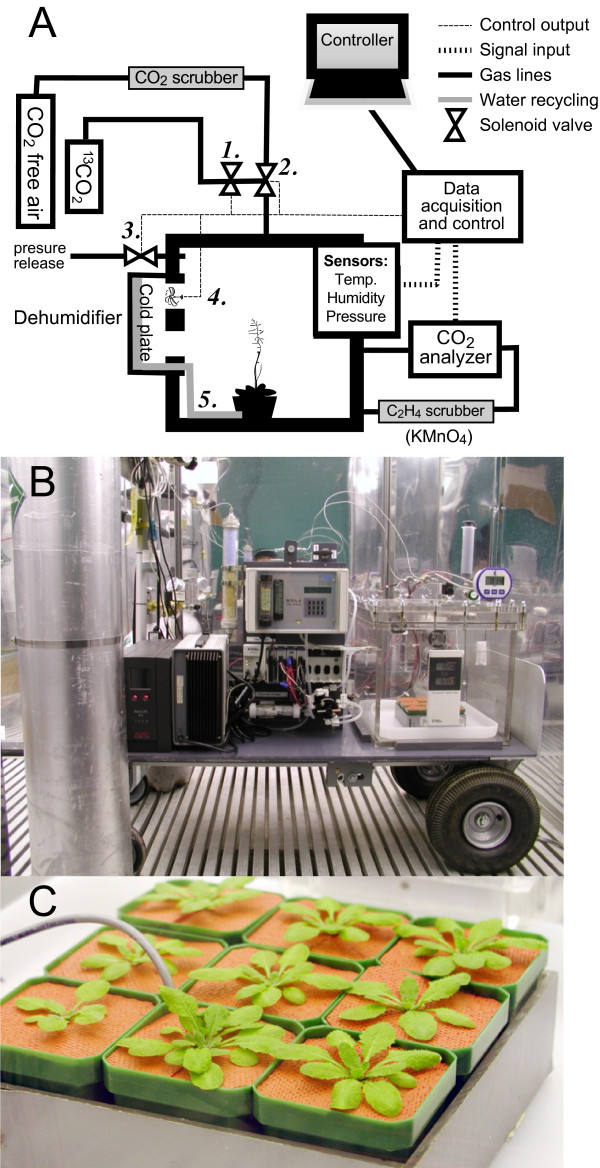
**Sealed enclosure for whole-plant labeling with ^13^CO_2_**. (A) Diagram of the system, including in-line gas flow control, continuous CO_2 _measurement, Peltier-based dehumidifier, ethylene scrubber, sensors for temperature, humidity and enclosure pressure and data acquisition and control devices. (B) Photograph of the system placed on its movable cart. The enclosure (30.4 cm × 30.4 cm **× **30.4 cm) shown is in its primary single cube configuration. An additional cube can be added on the top of the first cube to increase the total growth overhead space. (C) Three-week-old *Arabidopsis *seedlings grown in the enclosure from seed in 99 atom% ^13^CO_2_.

Our interest in using stable isotope labeling coupled with LC-MS/MS for determination of protein and metabolite turnover necessitated the construction of an automated and versatile ^13^CO_2 _labeling enclosure. The system we designed consists of a closed growth box assembled using predominately commercially available components. The enclosure itself and the housing for the Peltier-based dehumidifier were constructed in the university shop from Plexiglass^® ^acrylic sheets. Figure 1A and B show a schematic and photographic image, respectively, of the completed system. ^13^CO_2 _labeling using the system has been successfully tested by growing *Arabidopsis *as shown in Figure 1C. The controlled growth environment can accommodate 25 *Arabidopsis *plants seedlings grown hydroponically with a maximum head-space volume of ~50 L.

Initial tests of this system with *Arabidopsis *plants revealed that even very young seedlings (< 5-day old) emitted sufficient ethylene to result in significant stunting of growth and aborted embryo development (data not shown). Potassium permanganate is a strong oxidizing agent and can readily oxidize ethylene into CO_2 _and H_2_O. It has been used to delay the ripening of fruits and extend the freshness of cut flowers and vegetables by removal of ethylene from the air. The inclusion of potassium permanganate-based adsorbent packets (Power Pellet sachets, Ethylene Control, Selma, CA) to the system completely overcame these ethylene-mediated growth and developmental consequences and enabled us to grow *Arabidopsis *plants from seed to seed.

The design of the enclosure provided an environment where available CO_2 _had a high ^13^C enrichment (~99%) for growing whole plants. This was accomplished by repeated purges of the complete system with CO_2_-free air followed by a cycle of equilibration with 300 ppm ^13^CO_2_, a second purge, and re-equilibration. This was routinely performed to ensure the lowest contamination of ambient CO_2 _in the enclosure prior to initiating labeling experiments.

### ^13^C enrichment of plant derived compounds obtained using the enclosure

To investigate the efficiency of ^13^CO_2 _labeling using this enclosure, the enrichments in individual amino acids and peptides derived from proteins were monitored using GC-MS and LC-MS/MS, respectively.

Amino acids serve as precursors to many metabolites, including proteins, primary metabolites, plant hormones and nucleic acids, and function as the main carriers for nitrogen metabolism. In plants, photosynthesis plays a vital role in amino acid synthesis as it does in the production of all carbon containing metabolites. Monitoring amino acid enrichment and turnover should thus provide an excellent indication of how completely plants are labeled with ^13^CO_2_. We employed a rapid and highly sensitive methyl chloroformate derivatization GC-MS method for amino acid profiling [34] which, due to the low mass of the added groups and the stability of the derivatized amino acids, was well suited for such isotopic enrichment studies. We first determined the total number of carbon atoms in most of the major fragments of the methyl chloroformate-derivatized amino acids by using unlabeled and fully labeled amino acid standards (Additional file 2). The molecular or, alternatively, major fragment ions (typically [M-59]^+^) of sufficient intensity were used to provide isotopomer distribution information for the enrichment calculations.

**Figure 2:**
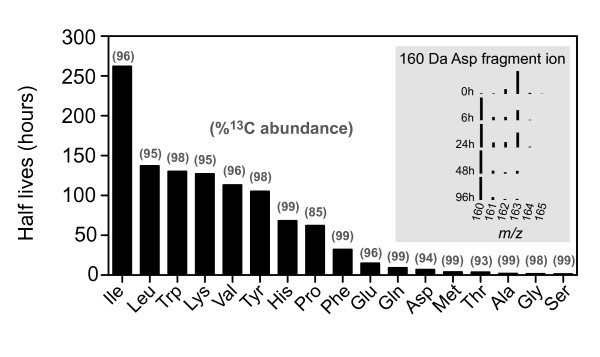
**Amino acid enrichment and turnover in the leaves of *Arabidopsis *plants labeled with ^13^CO_2_**. *Arabidopsis *seedlings were grown in the enclosure with 99 atom% ^13^CO_2 _for three weeks from seed then chased with ambient air (containing ^12^CO_2_) for different durations before sampling for amino acid analyses using GC-MS. Data from 3 amino acids, cysteine, asparagine and arginine, were not shown. The half-lives of amino acids were calculated by non-linear regression assuming a simple exponential decay process with no plateau. The percent ^13^C enrichment is given as the number listed parenthetically above the bars for each amino acid. A graph inside this figure shows the diminution of a heavy fragment ion (*m/z *163) and emergence of the light fragment ion (*m/z *160) of the derivatized [^13^C]-labeled aspartic acid (Asp) during the isotopic dilution period. The elemental composition of this specific fragment ion was determined to be C_6_H_10_NO_4_; but in the fragment only C_3_H_4_N_1 _originated from the amino acid and the remainder of the elements were from the derivatization reagent.

In order to measure amino acid turnover, after an initial ^13^CO_2 _labeling period beginning at germination, [^13^C]-enrichment was monitored immediately after removal from the ^13^CO_2 _enclosure and at various periods of growth in ambient air (with ^12^CO_2_) to allow dilution of the isotope pools. Samples of [^13^C]-labeled *Arabidopsis *leaves were harvested at least 4 h after the start of the light period to reduce major metabolic changes due to the diurnal cycle [19]. No significant differences in plant morphology were observable throughout the three-week growth period between plants grown inside and outside the [^13^C]-enclosure (using otherwise identical conditions, in the same walk-in growth chamber). Of 17 amino acids monitored, 9 amino acids, including serine, glycine, alanine, methionine, glutamine, histidine, tyrosine, phenylalanine and tryptophan, were essentially fully enriched (> 98%) after ^13^CO_2 _labeling from the time of seed germination (Figure 2). The remaining observable amino acids showed enrichments of >93%, except for proline, which had only 85% enrichment.

Amino acid half-lives in *Arabidopsis *leaves were determined using changes in isotopic distributions observed in mass spectra of labeled amino acid ions monitored over time. An example (aspartic acid, *m/z *160) of just such a time course is illustrated in Figure 2. The specific fragment ion (*m/z *160) contains six carbon atoms but only three of these were derived from aspartic acid. The other three carbon atoms originate from the methyl chloroformate derivatizing reagent. As shown, after being fully labeled with ^13^C, the peak at *m/z *= 163 becomes the most abundant peak in the isotopic cluster. After isotopic dilution with ambient CO_2 _for 96 h, the most abundant peak shifts back to *m/z *= 160, which is the [^12^C]-monoisotopic ion of this specific Asp fragment. The rate at which the isotopic distribution shifted back toward a natural isotopic abundance distribution reflected the turnover rate of this amino acid. Figure 2 shows the half-lives of a total of 17 amino acids measured and fitted to the equation for first order exponential decay. Among the amino acids, glycine, serine and alanine had the shortest half-lives (< 2 h). Glutamic acid, glutamine and aspartic acid, which are involved in nitrogen assimilation, had medium turnover rates that ranged from 7-15 h. Several amino acids had half-lives of over 4 d, including tyrosine, valine, lysine, tryptophan and leucine; isoleucine had the longest half-life. It is worth noting that the time course of isotope dilution was found to be biphasic in many cases. The carbon partition was initially fast, but then slowed down dramatically (data not shown). This phenomenon might be due to the recycling of labeled carbon for the newly synthesized amino acids followed by a slower phase approaching equilibrium after the initial linear dilution phase. In addition, amino acids might be present in multiple pools, which exhibit differing rates of exchange between pools. This biphasic isotopic dilution phenomenon has been described previously using ^13^CO_2 _in plants and with highly-enriched water (*δ*^2^H = 340 ± 1% or *δ*^18^O = 15.0 ± 0.1%) in animals [19,35].

### Protein enrichment and turnover measurement using the enclosure

**Figure 3:**
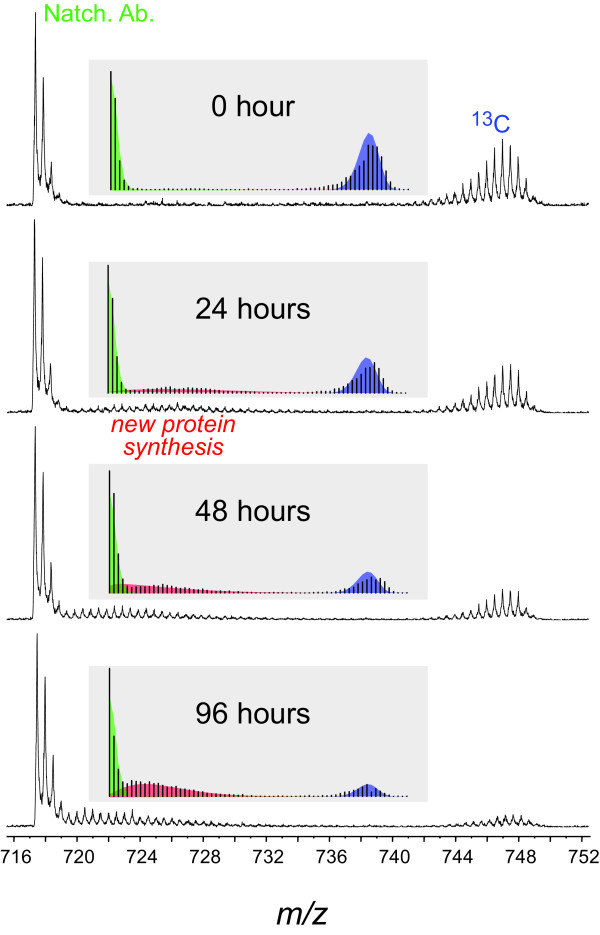
**Protein turnover demonstrated in mass spectra for a peptide from [^13^C]-labeled ATP synthase CF1 β-subunit**. *Arabidopsis *plants were grown with ^13^C-carbon dioxide in the enclosure for three weeks from seed then transferred to ambient air for 0 h, 24 h, 48 h and 96 h before the leaves were harvested for total protein extraction. Unlabeled proteins were added as a 'spike' to the [^13^C]-labeled samples before gel electrophoresis, protein band isolation, in-gel trypsin digestion and LC-MS/MS analysis. The observed spectra were fitted with three β-binomial distributions: natural abundance (green); newly synthesized peptide (red); and old peptide (blue) distributions shown for each spectrum in the insets. Sample spectra of the tryptic peptide from ATP synthase CF1 β -subunit (FVQAGSEVSALLGR, C_63_H_104_N_18_O_20_) show the disappearance of the ^13^C-labeled peptide over time. This peptide was doubly charged with a monoisotopic *m/z *of 717.391. In addition to a shift in the fractional isotopic abundance newly synthesized peptide (red) with time, the distribution abundance ratios of the newly synthesized peptide (red) and old peptide populations (blue) increase with time.

As discussed above, an important goal of this research was to develop a high throughput method to measure protein turnover using whole plant stable isotope labeling via LC-MS/MS on a proteomic scale. A protein turnover measurement using this enclosure with ^13^CO_2 _was demonstrated as shown in Figure 3. To assist the identification of partially labeled peptides in the raw MS/MS using a standard search algorithm, unlabeled proteins were added to [^13^C]-labeled proteins in a 1:4 ratio (unlabeled to labeled) before being separated by SDS-PAGE. A predominant protein band around 52 kDa containing mostly Rubisco large subunit was excised and subjected to in-gel trypsinization prior to LC-MS/MS analysis. Several proteins including Rubisco large subunit and ATP synthase CF1 β-subunit were identified in these samples with multiple high confidence peptide assignments. We are interested in measuring the turnover of β-subunit of ATP synthase because we have previously observed this protein to be modified by the plant hormone indole 3-acetic acid in other plant species [36]. Once unlabeled peptides from this protein were identified, both unlabeled and labeled peptides were confirmed as coeluting sets of isotopic distributions by linear correlation of extracted isotopic channels within known retention time windows.

Figure 3 shows the changes in isotopic distributions for a peptide of *Arabidopsis *ATP synthase CF1 β-subunit (FVQAGSEVSALLGR, C_63_H_104_N_18_O_20_) as they occur in discreet time points after switching from growth using ^13^C-labeled to unlabeled carbon dioxide. At time 0 only natural abundance (from the spike) and fully-labeled peptide distributions were present in the spectra. The [^13^C]-enrichment of the labeled peptide was estimated to be 93atom%. Twenty-four hours after plants were transferred to ambient air, an additional peptide distribution appeared. For this peptide the new distribution was clearly visible above noise following 24 hours of growth under unlabeled CO_2_. A pronounced shift in the isotopic distribution of this newly synthesized peptide toward natural abundance can also be observed over time and can be described using the rate of change in *fractional isotopic abundance *for this distribution. Perhaps a more important measurement with respect to protein turnover kinetics is the *distribution abundance ratio *of old peptide (synthesized prior to label change) to new peptide (synthesized after label change). This value can be measured by taking the ratios of modeled distributions for the old peptide (envelope on the right) and the newly synthesized peptide (envelope on the left next to the spiked natural abundance) and may be expected to decrease following first order kinetics.

When isotopic peak intensities are modeled as mixed β-binomial distributions, the observed spectra can be fitted and parameterized by a maximum likelihood estimate (MLE) into three isotopic distributions: natural abundance, newly synthesized peptide and old peptide distributions as highlighted by green, red and blue colors, respectively, in the insets of each spectrum. The mass shift of newly synthesized peptide distribution and the relative abundance of newly synthesized peptide and old peptide distributions can be derived from the fitted distribution parameters at each time point. As expected, we found that the *distribution abundance ratio *of newly synthesized peptides decreased following first order kinetics and could be fitted by non-linear regression to calculate half-life. However, the kinetics of fractional isotopic abundance of the newly synthesized peptide distribution seemed to be biphasic with a fast initial phase followed by a slow phase. In theory, the fractional isotopic abundance kinetics reflects a combination of amino acid turnover, protein turnover as well as the recycling of label compounds in the system. The half-life of *Arabidopsis *ATP synthase CF1 β-subunit in *Arabidopsis *leaves was calculated to be 57 hours using distribution abundance ratio measurements from 5 peptides (Additional file 3). The entire data analysis procedure has been implemented in R code for high-throughput calculations (further information available at website: http://www.proteinturnover.umn.edu/).

## Discussion

While most intact plants are limited in their capacity to absorb and utilize chemically complex labeled nutrients, they can be provided with chemically simple labeled nutrients (nitrate, carbon dioxide, water, phosphate etc.) to accomplish ubiquitous metabolic incorporation of isotopically labeled elements during the growth process [37]. While it is relatively easy to label many plants to high enrichment of ^15^N by simply providing labeled nitrogen nutrients in hydroponics media, [^13^C]-labeling from ^13^CO_2 _is much more difficult because of the need for atmospheric gas control. Other means of [^13^C]-labeling have been accomplished as described earlier and from non-volatile carbon sources such as glucose, but these methods result in rather extreme physiological perturbations that may be confounding for many experimental questions. In many cases, especially in protein analysis, [^15^N]-labeling is ideal, but for the analysis of metabolites, where nitrogen is not always present, or for investigations where nitrogen inputs cannot be modified as in nitrogen starvation, [^13^C]-labeling is required. For those situations where ^15^N is not a useful label we have developed a ^13^CO_2 _growth enclosure for propagation of *Arabidopsis *with ubiquitous carbon labeling to a high atom% enrichment of ^13^C.

Introduction of isotopic labels into biological systems can accomplish three fundamentally different types of experimental objectives: 1) Signal enhancement (e.g. radiolabeling, [^15^N]- or [^13^C]-labeling for NMR); 2) Molecular flux measurement (by following changes in isotope composition over time following changes in labeling inputs); and 3) Mass resolvable internal control (chemically identical but isotopically distinct samples are combined prior to extraction and mass spectral analysis). For signal enhancement, it is often not necessary or desirable to have high atom% isotope enrichment as this may result in undesirable excessive radiation or spin coupling in NMR. In contrast, for flux analysis or for relative quantification via mass spectrometry it is usually highly advantageous to be able to label molecules to near 100% atom substitution. To ensure a significantly high [^13^C]-enrichment of the plant material, the enclosure was designed to allow for the complete purging with CO_2 _free air. We found that the circulation of ^13^CO_2 _(300 ppm) during the purging cycle, before the start of the labeling experiments, helped increase the [^13^C]-enrichment of plants grown in the enclosure due possibly to the exchange of surface-bound and residual free ^12^CO_2 _in the system with ^13^CO_2_. This purging step was typically performed before seed germination, but could be used at any additional steps in the growth process as well. Especially for seeds with large nutrient stores, the enclosure could be purged at various times after germination to remove ^12^CO_2 _contamination produced during respirative consumption of seed carbon stores during the earliest stages of plant growth. Such a purge of a growing system would be expected to be best conducted just prior to the transition from dark to light in the photoperiod cycle so that the products of respiration are cleared prior to initiation of new photosynthetic carbon assimilation [19]. With our current design we noted when using the CO_2_-free air inlet, that the relative humidity could drop to as low as 40% with the introduction of the dry gas into the enclosure during purging steps. This drop may be avoided either by simply pre-humidification of the air, as with a gas sparger or alternatively, as we have found effective, by using only short purges (30 min) with dry air thus avoiding drought stress at the sensitive seedling stages of growth.

In plants, ^13^CO_2_, unlike [^13^C]glucose [21], should rapidly label cellular carbon with little discrimination at high enrichments [18,19,25] because CO_2 _enters the plant metabolic systems via photosynthesis where carbon backbones of all organic compounds are synthesized. Transfer from labeled to unlabeled environments should rapidly change the form of fixed carbon entering into the plant's metabolic system [19]. In addition, consistent with previous studies [18,25], the high percentage labeling with ^13^CO_2 _procedures yielded very clean mass spectral data making analysis of amino acid and protein turnover more efficient and accurate.

There are two important potential concerns regarding the use of ^13^CO_2 _for labeling that must be considered. First, since the plants will be completely labeled with ^13^C, the extent of carbon recycling will be an issue, especially under conditions where a dark period is required in the experimental protocol or where very young seedlings are used. In a series of amino acid turnover experiments (data not shown) as well as protein turnover experiments (Additional file 3), we observed dilution plateaus at various levels for most of the amino acids and peptides, suggestive of a significant level of carbon recycling. A similar observation of a plateau for various metabolites had been reported previously [19]. Second, both the cost and the limitations of the growth environment are important concerns since these may limit the application of the methods in specific cases. While seedling data should be possible to obtain using seeds produced on plants raised in the ^13^CO_2 _environment, changing the carbon isotopic composition during seedling growth might be a challenge due to the extensive use of stored reserves. Potentially either the seedlings could be grown on labeled sugars or the seedlings could be shifted to continuous autotrophic conditions to decrease the influence of stored seed reserves. Previously, we showed both the utility and limitations of using ^2^H_2_O for measurement of protein turnover in young seedlings [38].

Early seedling growth heavily depends on stored nutrients in seed endosperm including carbon resources. This mostly ^12^C carbon source in endosperm is very likely to cause reduction of final [^13^C] enrichment in plants. We found that *Arabidopsis *in this study as well as tomato plants (unpublished data) could be highly [^13^C] enriched within a month of growth in the enclosure regardless of the stored carbon resources in their endosperms and without intensively purging the enclosure after seed germinated. These results were likely due largely to the dilution effect of the high ratios of final fresh weight over seed weight in both plant species. However, for plants with larger carbon capacity within the seed, such as corn, rice and soybean, the residual endosperm for monocots or the cotyledons on dicots will likely need to be removed from the seeds prior to planting or from young seedlings to maximize the enrichment achieved. Such extra caution has been taken previously in rice labeling with ^13^CO_2 _and has been shown to increase [^13^C] enrichment [19].

## Conclusion

We have designed, tested and described an open source ^13^C-labeling growth environment for *Arabidopsis*. Our testing results demonstrate that this system provides an excellent general labeling system for *Arabidopsis *and allows minimal alteration of the plant due to the labeling procedure *per se*. In addition to being used for turnover or flux studies, the highly enriched protein and metabolite fractions from plants grown under ^13^CO_2 _can be used for quantitative studies of protein and metabolite levels in plants. This system is highly adaptable and can be readily modified for a variety of research procedures that require an enclosed growth environment. The software that controls the system is readily available and easily modified with tools provided with the software package.

## Materials and methods

### Chemicals

Carbon dioxide (^13^CO_2_, isotopic purity 99atom% ^13^C, <1.5atom% ^18^O) was purchased from Cambridge Isotopes (Andover, MA, USA) in a 10 L lecture bottle. CO_2 _free air (< 1 ppm CO_2_, 25% oxygen with a balance of nitrogen) was purchased from Minneapolis Oxygen Company (Minneapolis, MN, USA). The amino acid standard mixture was purchased from Thermo Scientific-Pierce (Rockford, IL, USA) and the [^13^C]-labeled algal amino acid mixture was purchased from Cambridge Isotope Laboratories. All other chemicals were obtained from Sigma-Aldrich (St Louis, MO, USA) at the highest available purity.

### Plant Growth and Labeling Conditions

*Arabidopsis thaliana *Col-0 seeds were surfaced sterilized using 70% ethanol for 1 min followed by 10% bleach containing 0.1% TritonX-100 for 20 min. After being washed with de-ionized water three times, seeds were sown on pre-rinsed rockwool plugs (3.75 cm × 3,75 cm, Grodan, Milton, ON, Canada), and covered with silicone rubber mats (4.7 cm × 4.7 cm, 8608K151, Extreme-Temp Textured Silicone Foam Rubber, 3.175 mm thick, ordered from McMaster-Carr, Robbinsville, NJ, USA) to restrict algal growth. Half strength Gib hydroponic medium [39] was used for growing the *Arabidopsis *in the enclosure. To minimize dissolved ^12^CO_2_, the medium was degassed using a helium spurge at 60°C for 30 min, capped, and then cooled to room temperature before being injected into the automated enclosure system. The growth parameters for *Arabidopsis *were 16 h light/8 h dark with 23°C during the day/18°C night air temperature, a constant 65% relative humidity, and a maximum light intensity of 100 μmol m^-2 ^s^-1^. The lighting was provided by a combination of fluorescence lamps (F96T12 CW VHO, Phillips, Andover, MA, USA) and incandescence light bulbs (40 W frost, Phillips). The internal atmospheric pressure was maintained at 2 kPa above ambient. In order to remove ambient CO_2 _from the enclosure after it was sealed, the enclosure was purged with CO_2 _free air until CO_2 _levels went down to <5 ppm, then ^13^CO_2 _was injected under software control into the enclosure until the levels reached 400 ppm. Air was allowed to circulate throughout the entire system, including the attached tubing, for 1 h then the system was purged again two additional times. After the system was thoroughly purged, the ^13^CO_2 _level was kept at 600 ppm for the duration of the experiment. Dilution with ^12^CO_2 _after ^13^CO_2 _labeling was achieved by exposure of three-week-old labeled plants to the ambient atmosphere in the same walk-in growth chamber that housed the ^13^C-enclosure, with height adjustments to maintain the same light intensity.

### Enclosure Construction

#### Hardware Setup

This growth enclosure was built from Plexiglass^® ^acrylic sheets (1.27 cm thickness) and had a two-level design (2 cube-shapedboxes total of 30.4 cm × 30.4 cm × 30.4 cm, volume = ~54 L). This design permitted a single enclosure to be used for routine work and the height doubled for experiments where flowering and seed production were to be studied. Viton^® ^gaskets (2 mm thick) were used as the sealing material between the two enclosures and to seal the lid because of its excellent chemical resistance and low gas permeability. A small Plexiglass^® ^acrylic enclosure with a thermoelectric cold plate (CP-031, TE Technology, Traverse City, MI, USA) installed on the back side of its walls was connected to the main enclosure by one inlet acrylic tube (5.08 cm, OD) and one outlet aluminum tube (5.08 cm, OD), as a heat exchanger for the return air. The cold plate was controlled by a temperature controller (TC-48-20, TE Technology) and was maintained at 10°C. This cold box is used to control the humidity of the main enclosure by circulating air from the main enclosure using a software controlled small fan (3.81 cm × 3.81 cm, 12 V) onto the cold plate such that the excess moisture in the air condensed on the cold surface. The detailed design of this dehumidifier can be found in the user's manual (see Additional file 4). Two software controlled solenoid gas valves were installed on the lid of the enclosure for inputs of CO_2 _gas and CO_2_-free air. One was a proportional two-way solenoid (EV-P-10-0925, Clippard Minimatic, Cincinnati, OH, USA) for controlling CO_2 _gas via a pulse-width modulator (Si5HyUdMC2-30 V-2 × 20A, Signal Consulting, Edgewater, MD, USA). The other was a three-way solenoid valve (EVO-3-24, Clippard Minimatic) that served as a switch between ^13^CO_2 _gas and CO_2 _free air. When energized, it switches to the CO_2 _free-air port for enclosure purging or maintaining enclosure pressure. In addition, a two-way solenoid valve that functions as a pressure relief valve (MME-2PDS, Clippard Minimatic), a pressure sensor (DPG1000DAR35KPAG-1N-I-CC, Cecomp Electronics, Libertyville, IL, USA) and a humidity/temperature sensor (HX94ACW, Omega Engineering, Stamford, CT, USA), were all installed on the lid. For injection of growth medium and withdrawing liquid from the growth tray, another needle valve (B-4JN2, Nupro, Willoughby, OH, USA) was installed on the lid. A standard luer-lock female fitting (McMaster Carr) was soldered to one end of the valve that faces outside the chamber to accept a 60 mL luer lock syringe (BD, Franklin Lakes, NJ, USA). Also, a standard luer-lock male fitting (McMaster Carr) was soldered to the other side of the valve to which a hypodermal needle (gauge 10; McMaster Carr) with a standard luer-lock female fitting was connected. The needle was long enough to slightly touch the bottom of the tray. Two small 12 V DC fans (5 cm × 5 cm) were also installed on the inside of the lid to provide air circulation within the enclosure. A CO_2 _analyzer (WMA-4, PP Systems, Amesbury, MA, USA) was located outside the enclosure and was connected via Bev-A-Line IV tubing (0.64 cm OD/0.32 cm ID, US Plastic Corporation, Lima, OH, USA). The CO_2 _analyzer housed a pneumatic diaphragm pump to continuously circulate air at a flow rate of 1 L/min from the growth enclosure through a small air chamber inside the analyzer where the CO_2 _sensor was located. After measurement, the air passed through a custom ethylene scrubber column (14 cm ×2.3 cm ID glass tube with Teflon compression fitting caps) containing five sachets of potassium permanganate-coated × pellets (2 × 9 g sachets, Ethylene Control) before flowing back to the main enclosure. A manual stack 4-way valve (224-X-PP-ALL-MS2, EVSCO, Libertyville, IL, USA) was used to direct the air coming out from the CO_2 _analyzer to either the ethylene scrubber or a bypass route, which allowed easy replacement of ethylene scrubber bags in the column while an experiment was in progress. After replacement, the scrubber column was purged with CO_2 _free air that was directed by a manually operating 3-way valve (H6800 SSL1/16PST, HAM-LET, Solon, OH, USA) located at the top of the CO_2 _analyzer. CO_2 _free air containing less than 1 ppm CO_2 _was run through a soda lime based CO_2 _scrubber (PP Systems, Amesbury, MA, USA) before being injected into the enclosure for purging purposes and/or for maintaining the slightly positive pressure of the enclosure. To prevent excessive CO_2 _gas addition beyond desired concentrations, the flow rate of CO_2 _gas was controlled by a needle valve (SS-SS2-VH, Swagelok, Chaska, MN, USA) set at the lowest rate. The outlet pressure of ^13^CO_2 _was controlled by the regulator (Y11-L244ALB, Airgas, Savage, MN, USA) that was attached directly to the lecture bottle. A pressure of ~10 psi was found to be ideal for optimal control of the ^13^CO_2 _flow rate. The CO_2 _free air flow rate during plant growth was controlled only by the restriction of ~100 cm of the attached 1.59 mm OD/0.51 mm ID stainless steel tubing when outlet pressure of the gas regulator (Y12-244 D, Airgas) was set at 100 psi. During the purging of the enclosure, the pressure was increased to 120 psi.

#### Power supplied to the system

A 12 V DC power supply (Model 1316, Global Specialities, Wallingford, CT, USA) was used to operate the thermoelectric cooler (cold plate), 3 small fans, and the PWM controller for the proportional solenoid. A 24 V DC power supply (Model 6216B, Agilent/HP) as used to control all other solenoid valves, temperature/relative humidity sensor and enclosure pressure sensor. The power supplies, the laptop computer (Toshiba Satellite M 115-S3094) that served as the controller, the data logger, as well as the CO_2 _analyzer are all directly plugged into a backup power system (Back-UPS RS 1500VA LCD 120 V; APC, Kingston, RI, USA).

#### System Control Setup

The current signal values from all sensors are acquired by a data acquisition device, NI Compact DAQ (cDAQ-9172, National Instruments) using an analog current input module (NI 9203, National Instruments) and compared to set points by a virtual control program (see Additional files 1 and 5) written in LabView (version 8.2 or above, National Instruments) on the notebook computer/controller. Output signals were triggered by the controller to turn on a relay module (NI 9481, National Instruments) on the Compact DAQ for the control of the CO_2_-free air solenoid, the fan in the dehumidifier, and pressure relief solenoid, until the values reached their set points. A proportional-integral-derivative (PID) control loop was used to control the solenoid valve for CO_2 _gas in a proportional manner. The voltage signal generated by a voltage signal module (NI 9263, National Instruments) on the Compact DAQ was transmitted to a pulse-width modulator where the signal was translated into power load to the solenoid valve. In addition, tuning parameters were also programmed into the control loop to deal with the delay in the report of CO_2 _levels in the enclosure due to the distance between the injection site and the CO_2 _sensor. To allow correct data acquisition and PID control using the Labview program provided, both the Compact DAQ data logger driver (NI DAQmx 8.2 or above, National Instruments) and the PID control tool kit (National Instruments) were also installed to the laptop computer/controller.

The enclosure pressure was maintained by a control loop in the program that compared actual pressure with the set minimum pressure and energized the air solenoid via a relay switch to inject enough CO_2_-free air into the system to maintain the set pressure. To return the enclosure pressure from an overpressure value, the pressure relief valve was energized by another relay switch. The enclosure pressure was kept at 2 kPa to prevent ambient CO_2 _from entering the enclosure. The control loop also allowed for enclosure purging by continuously opening the pressure relief valve until the purge was complete. While purging, the enclosure pressure was set at 1 kPa so that the pressure relief valve would remain continuously open during the purging process.

The fan inside the dehumidifier was activated by a corresponding relay on the Compact DAQ when the enclosure humidity became higher than the set point. The air in the main enclosure was drawn into the dehumidifier and excess moisture condensed on the surface of the cold plate and was returned by gravity to the hydroponics reservoir.

No lighting or temperature controls were integrated into the system as it was designed to operate within a walk-in growth chamber where lighting and temperature were independently regulated. We found that the acrylic used for the enclosure construction filtered out wavelengths shorter than 389 nm but did not absorb visible light wavelengths necessary for plant growth (Additional file 6). Total light intensity, however, was reduced approximately 16%. Thus, it is highly recommended that light intensity be carefully measured prior to each experiment and balanced against controls growing outside the enclosure by adjustments in the elevation of the control plants.

### Amino Acid Purification and Derivatization

Amino acid analysis was carried out according to Chen *et al*. [34]. Tissue samples of approximately 50 mg fresh weight were excised from *Arabidopsis *seedlings, transferred to microcentrifuge tubes, weighed and frozen in liquid nitrogen before storage at -80°C. Frozen tissues were ground in 1 mL of 10 mM HCl with 10 μL of methionine sulfone or stable isotope labeled amino acids (20-100 μg/mL) as internal standard using a bead grinding mill (5 min at frequency 25 s^-1^, MixerMill, Qiagen/Retsch Model MM330, Valencia, CA, USA). Samples were then centrifuged at 14,000 × *g *for 5 min. SCX SPE columns (100 mg resin, Grace, Deerfield, IL) were first wetted with 2 mL of distilled water three times using a vacuum manifold. The supernatants of the samples were transferred to the column and slowly drawn through. After sample loading, the columns were washed two times with 2 mL of a methanol/water mixture (8:1) and then the amino acids were eluted with 0.25 mL of 1:1 (v/v) 8 M NH_4_OH:methanol. A 50 μL aliquot of the analyte was transferred to a GC-MS vial insert and derivatized directly by mixing with 5 μL of pyridine and 5 μL of methyl chloroformate (MCF). To separate the MCF derivatives from the reactive mixture, 90 μL of chloroform and 90 μL of sodium bicarbonate solution (50 mM) were added and vortexed well. The bottom (chloroform) layer was transferred to a new GC insert containing few crystals of anhydrous sodium sulfate to dry the samples before they were used for GC-MS analysis.

### GC-MS Analysis of Amino Acids

All GC-MS analyses were performed using a Hewlett-Packard 5890 (GC)/5970 (MS) (Agilent) using electron impact (EI) ionization at 70 eV. The GC was equipped with a fused silica capillary column (HP-5MS, 30 m × 25 mm ID, 0.25 μm film thickness; Agilent J&W Scientific, Folsom, CA, USA). A 2 μL sample was injected in the splitless mode. The oven temperature was initially held at 70 °C for 3 min. Thereafter the temperature was increased using a gradient of 25°C/min until 280°C, followed by a temperature hold for 5 min. Helium was used as carrier gas and delivered at a constant flow rate at 1 mL/min during the run. The injector temperature was set at 240°C and the interface temperature was at 280°C. The mass spectra of the MCF derivatized amino acids and internal standards were obtained in either the full-scan or, alternatively SIM acquisition mode using a series of predetermined masses that changed based on the known elution time of specific sets of amino acids [34].

### Protein Extraction, Isolation and Trypsin Digestion

^13^C-labeled *Arabidopsis *leaves were harvested 0, 24, 48 and 96 h after the growth chamber was opened to ambient air. Plant material was ground in liquid N_2 _with a mortar and pestle and then total proteins were extracted and washed twice with ice-cold methanol containing a protease inhibitor cocktail (Roche, Indianapolis, IN, USA), then twice with ice-cold acetone. The protein pellets after centrifugation at 14,000 × g for 10 min were air-dried and resuspended in TE buffer containing 1% SDS. The protein concentration was estimated by the Bradford method [40] using a commercial kit from Bio-Rad (Hercules, CA, USA). Labeled protein samples were spiked with a known amount of the unlabeled protein (at 4: 1 ratio labeled to unlabeled) and separated by SDS-PAGE. Protein bands around 52 kDa corresponding to the molecular weight of the Rubisco large subunit, were excised manually after visualizing with colloidal Coomassie G-250 stain [41]. Excised bands were subjected to trypsin enzymatic digestion [42] on a ProPrep™ System (Genomic Solutions, Ann Arbor, MI, USA). Briefly, protein bands were subjected to two series of dehydration and hydration steps by the addition, incubation and removal of acetonitrile followed by the addition, incubation and removal of 25 mM NH_4_HCO_3_. Gel plugs were then reduced with 10 mM DTT/25 mM NH_4_HCO_3 _at 56°C for 30 minutes. The DTT solution was aspirated and a 55 mM iodacetamide/25 mM NH_4_HCO_3 _solution was added and the sample incubated for 30 minutes at room temperature. The iodacetamide solution was aspirated, followed by two series of dehydration and hydration steps as above. Protein bands were then subjected to tryptic digestion using 12 ng/μL trypsin (Sigma-Aldrich) in 25 mM NH_4_HCO_3_, 5 mM CaCl_2 _at 37°C for 10 h. The reaction was stopped with the addition of formic acid to a final concentration of 0.1% (v/v). Sample digests were manually aspirated and dispensed into 1.5 mL tubes with subsequent extraction by addition, incubation and removal to the respective tubes of 70% acetonitrile, 0.1% formic acid. All digested extracts were evaporated *in vacuo *(SC210A SpeedVac^® ^Plus, ThermoSavant, Asheville, NC USA), resuspended in LC-MS/MS loading buffer (98% H_2_O, 2% acetonitrile and 0.1% formic acid), and run on a QSTAR Pulsar *i *quadrupole-TOF MS system (Applied Biosystems, Foster City, CA, USA).

### LC-MS-MS analysis

Trypsin-digested peptides were separated and analyzed by a LC-MS/MS method described by Griffin *et al*. [43]. The LC system (LC Packings/Dionex, Sunnyvale, CA, USA) was interfaced with the QSTAR instrument (Applied Biosystems), which was equipped with a Protana (Odense, Denmark) nanoelectrospray source. Peptides (0.5 μg) were eluted with a linear gradient from 0-35% B (0.1% formic acid in a solution of 95:5 acetonitrile:water) over 45 min, followed by 35-80% B over 2 min, and held isocratic at 80% B for 10 min. Solvent A was 0.1% formic acid in 95:5 acetonitrile:water. Product ion spectra were collected in an information-dependent acquisition (IDA) mode, using continuous cycles of one full scan TOF MS from 400-1200 *m*/*z *(1 s) plus four product ion scans from 50-2000 *m*/*z *(2 s each). Precursor *m*/*z *values were selected starting with the most intense ion, using a selected quadrupole resolution of 3 Da. The rolling collision energy feature was used, which determines collision energy based on precursor *m/z *and charge state. Dynamic exclusion time for precursor ion *m*/*z *values was 60 s. MS/MS data were assigned using *ProteinPilot *(Applied Biosystems) and using the TAIR9 non-redundant *Arabidopsis thaliana *protein sequence database from *TAIR*. The list of identified peptides from confidently identified proteins was then saved in text format. Next, the original MS data in *WIFF *format was converted to *mzXML *format using the converter, mzWiff, from *The Trans-Proteomic Pipeline *developed at the *Institute for Systems Biology*. After the list of identified peptides and *mzXML *files were compileded they were then input into a program for the modeling algorithm written in R (described below) for the protein turnover calculation.

### Determination of ^13^C Enrichment

We adopted the method described by MacCoss *et al*. [44] for the estimation of ^13^C enrichment of compounds extracted from the ^13^CO_2 _labeled plants. The predicted isotope distribution was based on all natural isotope abundances with the exception of selected elements defined by the user as "enriched". For the enriched element(s) the isotope enrichment is varied from 0 to 100%. Each predicted isotope distribution was then correlated against the measured isotope distribution to find a best-predicted isotope distribution that is most representative of the experimentally measured isotope distribution using the Pearson correlation coefficient (*r*). The relative intensity for each peak in the predicted isotope distribution was calculated as described by Kubinyi [45].

A program implemented in R to accomplish these calculations is available from the authors upon request (further information is available at website: http://www.proteinturnover.umn.edu/).

### Determination of Amino Acid Turnover

The theoretical mass isotopomer distributions of 100% [^13^C]-labeled amino acid fragment ions were calculated according to the binomial distribution model as described previously [38,46,47]. The experimental mass isotopomer distributions of the ^13^C-labeled and unlabeled amino acid fragment ions were obtained by GC-MS and were used to calculate the relative isotope abundance (*Rt*) at each time, *t*, as a ratio of total net experimental fractional abundance of the mass isotopomers of the labeled ions and total net theoretical fractional abundance of mass isotopomers of 100% [^13^C]- labeled ions as shown in equation (1).

(1) $$R_{t}=\frac{\sum_{i=1}^{n}\left(EMi-SMi\right)\times i}{\sum_{i=1}^{n}\left(TMi-SMi\right)\times i}$$

In equation (1), *i *is the number of carbon atoms in any amino acid derived ion used for turnover calculation. In the mass spectrum, *i *provides the number of possible isotopic peaks appearing in roughly integer increments above the monoisotopic peak. This value was used as a normalizing parameter as its magnitude reflects the fractional contribution of ^13^C to ^12^C in each isotopic peak. To calculate *R_t_*, we first calculated the total net isotopic abundance of observed labeled amino acids by summing the normalized differences in fractional abundance for each peak of the distribution between the experimental samples *(EMi) *and the unlabeled (natural abundance) standards (*SMi*). Similarly, the total net isotopic abundance of 100% [^13^C]-labeled amino acids can be calculated by summing the normalized differences in fractional abundance for each peak of the distribution between the 100% [^13^C]-labeled amino acid *(TMi) *and the unlabeled standards (*SMi*). The isotopic peak distribution for unlabeled standards can be obtained from the experimental data or it can be generated from the combination of theoretical binomial distributions of each naturally occurring stable isotope in any given elemental composition; *TMi *is the theoretical fractional abundance of isotopic peaks occurring in the 100% labeled amino acid ions.

The value of *R_t _*changes over time as the amino acids are first prelabeled with ^13^C and are then repopulated by ^12^C. This shift in distribution occurs through normal intracellular amino acid metabolism following transfer from ^13^CO_2 _to ^12^CO_2_. Turnover rates can be estimated by nonlinear curve fitting of the plot of *R*_t _measured over multiple time points and fitted to an equation for exponential decay either without (Eq. 2) or with a plateau (Eq. 3) parameter.

(2) $$R_{\text{t}}=R_{0}e^{–kt}$$

(3) $$R_{\text{t}}=\left(R_{0}–\text{plateau}\right)e^{–kt}+\text{plateau}$$

*R_0 _*is relative isotopic abundance from Eq. 1 when *t *(time) is zero, meaning at the onset of dilution experiment. *Plateau *is relative isotopic abundance *R *at infinite times, *k *is the rate constant. Once *k *is computed, then the half-life of an amino acid can be computed as Eq. 4.

(4) $$t_{½}=\text{ln}\left(\text{2}\right)/k$$

The half-lives of amino acids shown in Figure 2 were calculated using Eq. 2 assuming no plateau. The algorithm has been implemented in a Windows Excel 2003 format for ease of use of this MIDA calculation.

### Determination of Protein Turnover

An algorithm implemented in R was developed by us to extract isotopic distribution information from raw MS data for multiple peptides identified by tandem MS. Then, the isotopic distributions were modeled by maximum likelyhood estimation using β-binomial distributions for: 1) spiked natural abundance, 2) newly synthesized peptide and 3) old peptide distributions. The workflow of the algorithm is described briefly as below. First, the algorythm was provided with a list of identified peptides (peptide amino acid sequence, and detected *m/z *and retention information) and the raw MS data in *mzXML *format. Then the number of carbon atoms were calculated for each peptide using the amino acid composition. Each carbon isotopic channel was assigned an *m/z *value calculated from the observed monoisotopic *m/z *value plus the ^13^C mass defect. Six additional channels were included to take account of the natural abundance of other isotopes (^15^N, ^2^H, ^18^O etc.). Extracted ion chromatograms were generated for each peptide at each isotope channel within a 5 min window centered on the retention time when the identifying MS/MS spectrum was triggered. This set of isotope abundances in each retention window for each peptide served as the data set for all analyses. Next, a linear regression analysis was performed for each isotope channel against the monoisotope channel for each peptide to reduce chemical noise and overlapping uncorelated peptide signals in the extracted spectra. Maximum likelihood estimation was performed to calculate the fractional isotopic abundance of the newly synthesied peptide distribution and the distribution abundance ratio of old to newly synthesized peptide distributions. Finally, half-life of each peptide and protein was calculated from changes in the distribution abundance ratios using non-linear-regression. Development and evaluation of algorithm is ongoing and a β-version of the web-based calculator and standalone software in R is available (further information at website: http://www.proteinturnover.umn.edu/).

## List of abbreviations

DAQ: data acquisition; DTT: dithiothreitol; GC: gas chromatography; ID: inner diameter; IDA: information: dependent acquisition; LC: liquid chromatography; MCF: methyl chloroformate; MIDA: mass isotopomer distribution analysis; MLE: maximum likelihood estimate; MS: mass spectrometry; MS/MS: tandem mass spectrometry; NMR: nuclear magnetic resonance; OD: outer diameter; PID: proportional integral derivative; PWM: pulse: width modulation; Rubisco: ribulose: 1,5: bisphosphate carboxylase oxygenase; SCX: strong cation exchange; SDS: sodium dodecyl sulfate (detergent); SDS: PAGE: sodium dodecyl sulfate polyacrylamide gel electrophoresis; SIM: selected ion monitoring; SPE: solid phase extraction; TAIR: The Arabidopsis Information Resource; TE: buffer containing 10 mM tris(hydroxymethyl)aminomethane and 1 mM ethylenediaminetetraacetate with pH adjusted to pH 8.0 by addition of HCl; TOF MS: time: of-flight mass spectrometry.

## Competing interests

The authors declare that they have no competing interests.

## Authors' contributions

WPC contributed to the design, construction and testing of the enclosure, development of methods for data analysis, analysis and interpretation of data and drafting of the manuscript. XYY participated in development of the methods for and in the analysis and interpretation of the data. GLH contributed to the design and construction of the enclosure. WMG conceived of the study and participated in analysis and interpretation of data, ADH participated in the design and testing of the enclosure, the development of methods for data analysis, and in the preparation of the manuscript. JDC conceived of the study, participated in its design and coordination and helped draft the manuscript. All authors read and approved the final manuscript.

## Supplementary Material

Additional file 1 **Customized software written in LabView**. The control code is shown in a graphical block diagram on which different function-nodes are connected graphically. The code is supplied as a png image, so that it can be pasted into an empty block in Labview 2009 (or any subsequent versions) to create a working block diagram. Click here for file

Additional file 2 **Amino acid analysis fragment ions**. Table of mass fragment ions of *N*-methoxycarbonyl amino acid methyl esters generated by 70 eV electron impact GC-MS analysis. Click here for file

Additional file 3 **Protein turnover first order decay curves**. The first order decay curves are shown for five independently derived tryptic peptides from ATP synthase CF1 β-subunit. The distribution abundance ratios of old peptide to newly synthesized peptide decreased over time and were fitted to a first order decay equation using non-linear regression. Click here for file

Additional file 4 **User's manual for the enclosure system**. Detailed user's manual describes set-up and operation of the enclosure. Several photographs are included at the end of the manual for reference. Click here for file

Additional file 5 **Screen shot of the control panel showing the user-friendly software control panel**. The desired enclosure humidity, pressure and CO_2 _level can be set easily on the panel. Users can also monitor enclosure humidity, temperature, pressure and CO_2 _level simultaneously. A software 'button' is included on the control panel for controlling the purging of the enclosure. Click here for file

Additional file 6 **Light spectrum in the enclosure with and without the acrylic lid**. The spectral photon distribution was measured with an Apogee Model SPEC-UV/PAR spectroradiometer. Inset shows complete spectral photon distribution from 200-800 nm. The lighting system in the walk-in growth chamber where the enclosure was placed consisted of both fluorescent and incandescent lights. Peaks observed in the photon distribution are typical mercury lines emitted from fluorescent light tubes. When the enclosure was covered with the Plexiglass^® ^acrylic lid, wavelengths shorter than 389 nm were filtered out but the enclosure lid would not absorb visible light wavelengths necessary for plant growth. The light intensities with and without the enclosure lid were measured at 158 and 188, respectively. Thus, the light intensity was ~16% reduced by the lid. Click here for file

## References

[B1] Schaefer J Kier LD Stejskal EO Characterization of photorespiration in intact leaves using C-13 dioxide labeling Plant Physiol 1980 65 254 259 10.1104/pp.65.2.254 16661170 PMC440307

[B2] Loreto F Pinelli P Brancaleoni E Ciccioli P ^13^C labeling reveals chloroplastic and extrachloroplastic pools of dimethylallyl pyrophosphate and their contribution to isoprene formation Plant Physiol 2004 135 1903 1907 10.1104/pp.104.039537 15286296 PMC520762

[B3] Cegelski L Schaefer J NMR determination of photorespiration in intact leaves using in vivo ^13^CO_2 _labeling J Magn Reson 2006 178 1 10 10.1016/j.jmr.2005.10.010 16289757

[B4] Calvin M The photosynthetic carbon cycle J Chem Soc 1956 1895 1915 10.1039/jr9560001895

[B5] Osmond CB Allaway WG Sutton BG Troughton JH Lüttge U Winter K Carbon Isotope Discrimination in Photosynthesis of CAM Plants Nature 1973 246 41 42 10.1038/246041a0

[B6] Schwender J Goffman F Ohlrogge JB Shachar-Hill Y Rubisco without the Calvin cycle improves the carbon efficiency of developing green seeds Nature 2004 432 779 782 10.1038/nature03145 15592419

[B7] Hutchinson CR Hsia MT Stephen CRA Biosynthetic studies with carbon-13 dioxide of secondary plant metabolites. Nicotiana alkaloids. 1. Initial experiments J Am Chem Soc 1976 98 6006 6011 10.1021/ja00435a038 965635

[B8] Kurilich A Britz S Clevidence B Novotny J Isotopic labeling and LCAPCI-MS quantification for investigating absorption of carotenoids and phylloquinone from kale (*Brassica oleracea*) J Agric Food Chem 2003 51 4877 4883 10.1021/jf021245t 12903939

[B9] Leake JR Ostle NJ Ignacio Rangel-Castro J Johnson D Carbon fluxes from plants through soil organisms determined by field ^13^CO_2 _pulse-labelling in an upland grassland Applied Soil Ecology 2006 33 152 175 10.1016/j.apsoil.2006.03.001

[B10] Kagawa A Sugimoto A Maximov TC Seasonal course of translocation, storage and remobilization of ^13^C pulse-labeled photoassimilate in naturally growing *Larix gmelinii *saplings New Phytol 2006 171 793 803 10.1111/j.1469-8137.2006.01780.x 16918550

[B11] Lee BR Jin YL Jung WJ Avice JC Morvan-Bertrand A Ourry A Park CW Kim TH Water-deficit accumulates sugars by starch degradation--not by de novosynthesis-- in white clover leaves (*Trifolium repens*) Physiol Plant 2008 134 403 411 10.1111/j.1399-3054.2008.01156.x 18785903

[B12] Rittenberg D Foster G A new procedure for quantitative analysis by isotope dilution, with application to the determination of amino acids and fatty acids J Biol Chem 1940 133 737 744

[B13] San Pietro A Rittenberg D A study of the rate of protein synthesis in humans: I. Measurement of the urea pool and urea space J Biol Chem 1953 201 445 455 13044814

[B14] Epstein E Cohen JD Bandurski RS Concentration and metabolic turnover of indoles in germinating kernels of *Zea mays *L Plant Physiol 1980 65 415 421 10.1104/pp.65.3.415 16661204 PMC440345

[B15] Baldi B Maher B Slovin J Cohen JD Stable isotope labeling in vivo of D and L tryptophan pools in *Lemna gibba *and the low incorporation of label into indole 3 acetic acid Plant Physiol 1991 95 1203 1208 10.1104/pp.95.4.1203 16668112 PMC1077673

[B16] Hedden P Modern methods for the quantitative analysis of plant hormones Annu Rev Plant Physiol Plant Mol Biol 1993 44 107 129 10.1146/annurev.pp.44.060193.000543

[B17] Barkawi LS Tam YY Tillman JA Pederson B Calio J Al-Amier H Emerick M Normanly J Cohen JD A high-throughput method for the quantitative analysis of indole-3-acetic acid and other auxins from plant tissue Anal Biochem 2008 372 177 188 10.1016/j.ab.2007.08.009 17889819

[B18] Römisch-Margl W Schramek N Radykewicz T Ettenhuber C Eylert E Huber C Römisch-Margl L Schwarz C Dobner M Demmel N Winzenhörlein B Bacher A Eisenreich W ^13^CO_2_ as a universal metabolic tracer in isotopologue perturbation experiments Phytochemistry 2007 68 2273 2289 17507062 10.1016/j.phytochem.2007.03.034

[B19] Huege J Sulpice R Gibon Y Lisec J Koehl K Kopka J GC-EI-TOF-MS analysis of in vivo carbon-partitioning into soluble metabolite pools of higher plants by monitoring isotope dilution after ^13^CO_2 _labeling Phytochemistry 2007 68 2258 2272 10.1016/j.phytochem.2007.03.026 17475294

[B20] Roessner-Tunali U Liu JL Leisse A Balbo I Perez-Melis A Willmitzer L Fernie AR Kinetics of labelling of organic and amino acids in potato tubers by gas chromatography-mass spectrometry following incubation in C-13 labelled isotopes Plant J 2004 39 668 679 10.1111/j.1365-313X.2004.02157.x 15272882

[B21] Ettenhuber C Radykewicz T Kofer W Koop HU Bacher A Eisenreich W Metabolic flux analysis in complex isotopomer space. Recycling of glucose in tobacco plants Phytochemistry 2005 66 323 335 10.1016/j.phytochem.2004.12.014 15680989

[B22] Kruger NJ Huddleston JE Le Lay P Brown ND Ratcliffe RG Network flux analysis: impact of ^13^C-substrates on metabolism in *Arabidopsis thaliana *cell suspension cultures Phytochemistry 2007 68 2176 2188 10.1016/j.phytochem.2007.03.033 17499825

[B23] Hegeman AD Schulte CF Cui Q Lewis IA Huttlin EL Eghbalnia H Harms AC Ulrich EL Markley JL Sussman MR Stable Isotope Assisted Assignment of Elemental Compositions for Metabolomics Anal Chem 2007 79 18 6912 6921 10.1021/ac070346t 17708672

[B24] Birkemeyer C Wagner C Erban A Kopka J Metabolome analysis: the potential of *in vivo *labeling with stable isotopes for metabolite profiling Trends Biotechnol 2005 23 28 33 10.1016/j.tibtech.2004.12.001 15629855

[B25] Giavalisco P Hummel J Lisec J Inostroza AC Catchpole G Willmitzer L High-resolution direct infusion-based mass spectrometry in combination with whole ^13^C metabolome isotope labeling allows unambiguous assignment of chemical sum formulas Anal Chem 2008 80 9417 9425 10.1021/ac8014627 19072260

[B26] Roessner U Luedemann A Brust D Fiehn O Linke T Willmitzer L Fernie AR Metabolic profiling allows comprehensive phenotyping of genetically or environmentally modified plant systems Plant Cell 2001 13 11 29 10.1105/tpc.13.1.11 11158526 PMC2652711

[B27] Fiehn O Kopka J Dormann P Altmann T Trethewey RN Willmitzer L Metabolite profiling for plant functional genomics Nat Biotechnol 2000 18 1157 1161 10.1038/81137 11062433

[B28] Desbrosses GG Kopka J Udvardi MK *Lotus japonicas *metabolic profiling. Development of gas chromatography-mass spectrometry resources for the study of plant-microbe interactions Plant Physiol 2005 137 1302 1318 10.1104/pp.104.054957 15749991 PMC1088322

[B29] Lisec J Schauer N Kopka J Willmitzer L Fernie AR Gas chromatography mass spectrometry-based metabolite profiling in plants Nat Protocols 2006 1 387 396 10.1038/nprot.2006.59 17406261

[B30] van Winden W Verheijen P Heijnen S Possible pitfalls of flux calculations based on ^13^C-labeling Metab Eng 2001 3 151 162 10.1006/mben.2000.0174 11289791

[B31] Loreto F Ciccioli P Cecinato A Brancaleoni E Frattoni M Fabozzi C Tricoli D Evidence of the Photosynthetic Origin of Monoterpenes Emitted by *Quercus ilex *L. Leaves by ^13^C Labeling Plant Physiol 1996 110 1317 1322 12226263 10.1104/pp.110.4.1317 PMC160926

[B32] Schaefer J Beard CF C-13 nuclear magnetic resonance analysis of metabolism in soybeans labelled by ^13^CO_2_ Plant Physol 1975 55 1048 1053 10.1104/pp.55.6.1048 PMC541763 16659207

[B33] Ostle N Ineson P Benham D Sleep D Carbon assimilation and turnover in grassland vegetation using an *in situ *^13^CO_2 _pulse labeling system Rapid Commun Mass Spectrom 2000 14 1345 1350 10.1002/1097-0231(20000815)14:15<1345::AID-RCM22>3.0.CO;2-B 10920353

[B34] Chen W-P Yang X-Y Hegeman AD Gray WM Cohen JD Micro-scale preparation of derivatized amino acids with methyl chloroformate for gas chromatography-mass spectrometry analysis J Chromatogr B 2010 878 2199 2208 10.1016/j.jchromb.2010.06.027 20663719

[B35] Cerling TE Ayliffe LK Dearing MD Ehleringer JR Passey BH Podlesak DW Torregrossa AM West AG Determining biological tissue turnover using stable isotopes: the reaction progress variable Oecologia 2007 151 175 189 10.1007/s00442-006-0571-4 17186277

[B36] Park S Cohen JD Slovin JP Strawberry fruit protein with a novel indole-acyl modification Planta 2006 224 1015 1022 10.1007/s00425-006-0287-z 16683161

[B37] Beynon RJ Pratt JM Metabolic labeling of proteins for proteomics Mol Cell Proteomics 2005 4 857 872 10.1074/mcp.R400010-MCP200 15849272

[B38] Yang XY Chen WP Rendahl AK Hegeman AD Gray WM Cohen JD Measuring the turnover rates of *Arabidopsis *proteins using deuterium oxide: an auxin signaling case study Plant J 2010 63 680 695 10.1111/j.1365-313X.2010.04266.x 20525007

[B39] Gibeaut DM Hulett J Cramer GR Seemann JR Maximal biomass of *Arabidopsis thaliana *using a simple, low-maintenance hydroponic method and favorable environmental conditions Plant Physiol 1997 115 317 319 10.1104/pp.115.2.317 9342857 PMC158488

[B40] Bradford MM A rapid and sensitive for the quantitation of microgram quantitites of protein utilizing the principle of protein-dye binding Anal Biochem 1976 72 248 254 10.1016/0003-2697(76)90527-3 942051

[B41] Candiano G Bruschi M Musante L Santucci L Ghiggeri GM Carnemolla B Orecchia P Zardi L Righetti PG Blue silver: A very sensitive colloidal Coomassie G-250 staining for proteome analysis Electrophoresis 2004 25 1327 1333 10.1002/elps.200305844 15174055

[B42] Shevchenko A Wilm M Vorm O Mann M Mass spectrometric sequencing of proteins silver-stained polyacrylamide gels Anal Chem 1996 68 850 858 10.1021/ac950914h 8779443

[B43] Griffin TJ Xie H Bandhakavi S Popko J Mohan A Carlis JV Higgins L iTRAQ reagent-based quantitative proteomic analysis on a linear ion trap mass spectrometer J Proteome Res 2007 6 4200 4209 10.1021/pr070291b 17902639 PMC2533114

[B44] MacCoss MJ Wu CC Matthews DE Yates JR Measurement of the isotope enrichment of stable isotope-labeled proteins using high-resolution mass spectra of peptides Anal Chem 2005 77 7646 7653 10.1021/ac0508393 16316172

[B45] Kubinyi H Calculation of isotope distributions in mass spectrometry. A trivial solution for a non-trivial problem Analytica Chimica Acta 1991 247 107 111 10.1016/S0003-2670(00)83059-7

[B46] Lee WN Byerley LO Bergner EA Edmond J Mass isotopomer analysis: theoretical and practical considerations Biol Mass Spectrom 1991 20 451 458 10.1002/bms.1200200804 1768701

[B47] Hellerstein MK Neese RA Mass isotopomer distribution analysis at eight years: theoretical, analytic, and experimental considerations Am J Physiol 1999 276 1146 1170 10.1152/ajpendo.1999.276.6.E1146 10362629

