# Supplementary figures and images for: An automated growth enclosure for metabolic labeling of Arabidopsis thaliana with 13C-carbon dioxide - an in vivo labeling system for proteomics and metabolomics research
Source: Proteome Sci. 2011 Feb 10;9:9. doi: 10.1186/1477-5956-9-9 (PMC3046907; doi:10.1186/1477-5956-9-9)

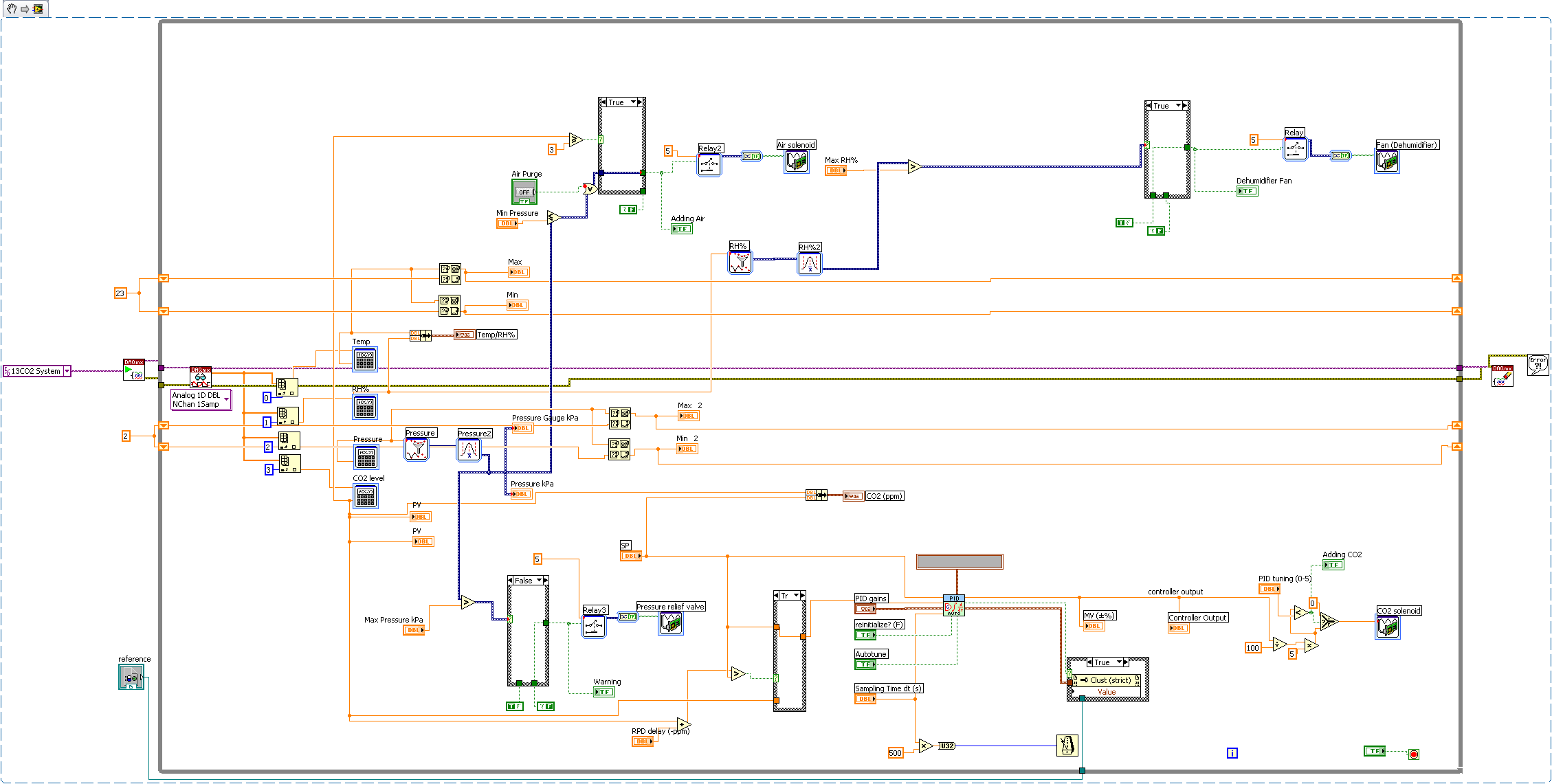

Supplement: Additional file 1 — Customized software written in LabView. The control code is shown in a graphical block diagram on which different function-nodes are connected graphically. The code is supplied as a png image, so that it can be pasted into an empty block in Labview 2009 (or any subsequent versions) to create a working block diagram. [file 1477-5956-9-9-S1.PNG]

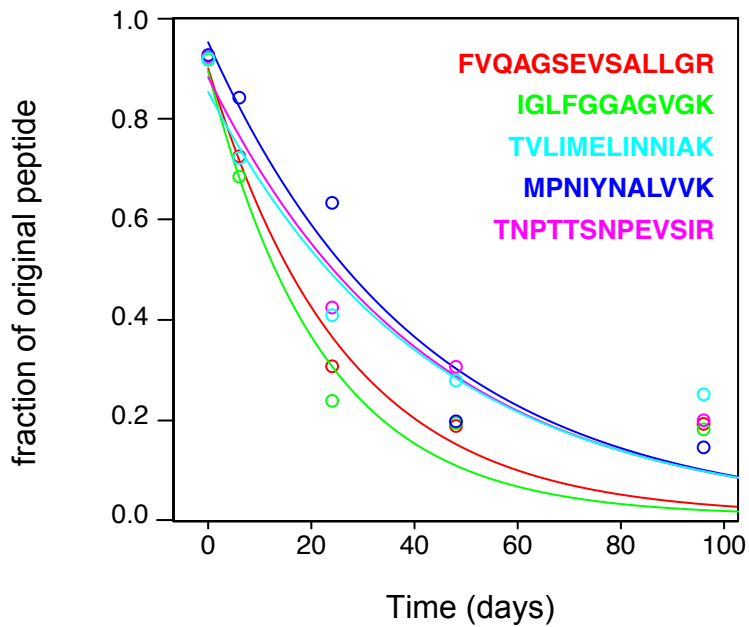

Supplement: Additional file 3 — Protein turnover first order decay curves. The first order decay curves are shown for five independently derived tryptic peptides from ATP synthase CF1 β-subunit. The distribution abundance ratios of old peptide to newly synthesized peptide decreased over time and were fitted to a first order decay equation using non-linear regression. [file 1477-5956-9-9-S3.PDF]

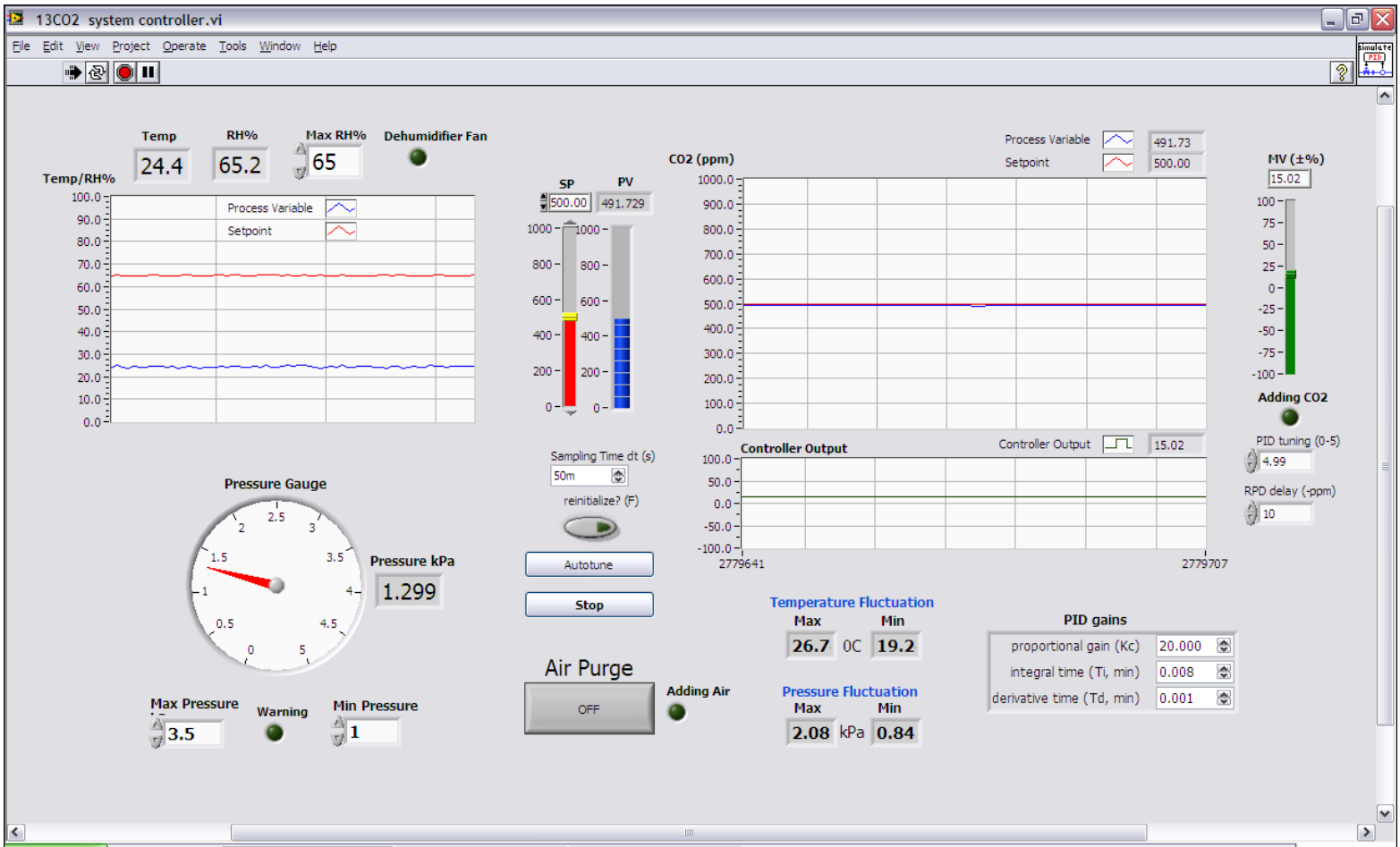

Supplement: Additional file 5 — Screen shot of the control panel showing the user-friendly software control panel. The desired enclosure humidity, pressure and CO2 level can be set easily on the panel. Users can also monitor enclosure humidity, temperature, pressure and CO2 level simultaneously. A software 'button' is included on the control panel for controlling the purging of the enclosure. [file 1477-5956-9-9-S5.PDF]

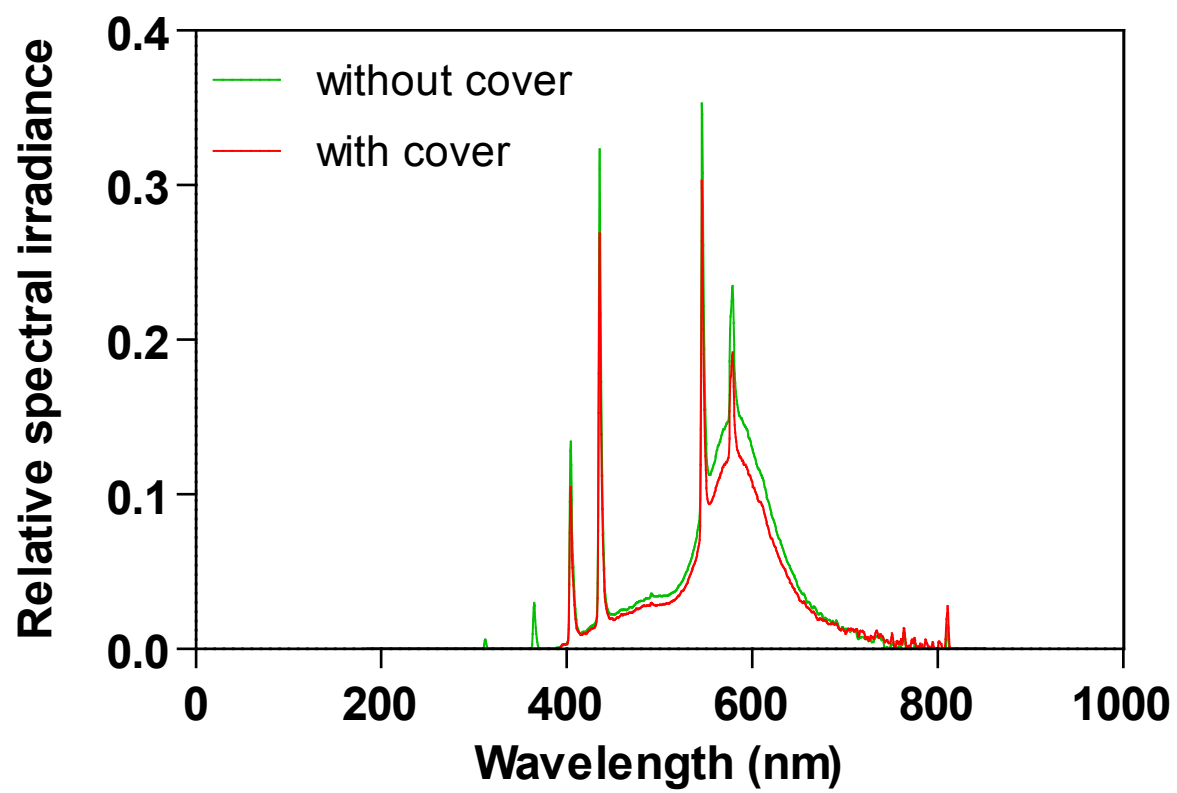

Supplement: Additional file 6 — Light spectrum in the enclosure with and without the acrylic lid. The spectral photon distribution was measured with an Apogee Model SPEC-UV/PAR spectroradiometer. Inset shows complete spectral photon distribution from 200-800 nm. The lighting system in the walk-in growth chamber where the enclosure was placed consisted of both fluorescent and incandescent lights. Peaks observed in the photon distribution are typical mercury lines emitted from fluorescent light tubes. When the enclosure was covered with the Plexiglass® acrylic lid, wavelengths shorter than 389 nm were filtered out but the enclosure lid would not absorb visible light wavelengths necessary for plant growth. The light intensities with and without the enclosure lid were measured at 158 and 188, respectively. Thus, the light intensity was ~16% reduced by the lid. [file 1477-5956-9-9-S6.PDF]
