# Supplementary material for: An automated growth enclosure for metabolic labeling of Arabidopsis thaliana with 13C-carbon dioxide - an in vivo labeling system for proteomics and metabolomics research
Source: Proteome Sci. 2011 Feb 10;9:9. doi: 10.1186/1477-5956-9-9 (PMC3046907; doi:10.1186/1477-5956-9-9)
Supplement: Additional file 2 — Amino acid analysis fragment ions. Table of mass fragment ions of N-methoxycarbonyl amino acid methyl esters generated by 70 eV electron impact GC-MS analysis. [file 1477-5956-9-9-S2.DOCX]

**Supplement Table ST1**

Mass fragment ions of *N*-methoxycarbonyl amino acid methyl esters generated by 70 eV electron impact GC-MS analysis

| Amino acid^a^ | RT (min) | Molecular ion | Major fragment ions (*m/z*) | Detected fragment^b^ (*m/z*) | Chemical formula^c^ | Elements originally from amino acid^d^ |
| --- | --- | --- | --- | --- | --- | --- |
| Gly | 6.01 | 147 | 88 | 147 | C5H9NO4 | C2H3N1O2 |
| Ala | 6.04 | 161 | 102,88 | 102 | C4H8NO2 | C2H5N1 |
| Val | 7.00 | 189 | 146,130,115,98 | 130 | C6H12NO2 | C4H9N1 |
| Leu | 7.52 | 203 | 144,115,102,88 | 144 | C7H14NO2 | C5H11N1 |
| Ile | 7.61 | 203 | 144,115,101,88 | 144 | C7H14NO2 | C5H11N1 |
| Thr-OH | 7.68  7.86 | 205 | 147, 115, 100 | 147 | C5H9NO4 | C2H3N1O2 |
| Pro | 7.93  8.28 | 187 | 128,84 | 128 | C6H10NO2 | C4H7N1 |
| Asn | 8.65  8.75 | 262 | 146, 127, 95 | 127 | C5H7N2O2 | C5H4N2 |
| Asp | 8.95 | 219 | 160,128,118,101,96,86 | 160 | C6H10NO4 | C3H4N1 |
| Ser-OH | 9.06 | 191 | 176,146,115,100 | 100 | C4H6NO2 | C2H3N1 |
| Gln | 9.54 | 276 | 141, 109, 82 | 141 | C5H5N2O3 | C4H4N2O1 |
| Glu | 9.69 | 233 | 201,174,142,114,98 | 174 | C7H12NO4 | C4H6N1O2 |
| Met | 11.05 | 221 | 142,128,115 | 221 | C8H15NO4S | C5H9NO2 |
| Cys | 11.37 | 192 | 176,158,146,132 | 192 | C6H10NO4S | C3H4NO2 |
| Phe | 11.90 | 237 | 178,162,146,131,103,91 | 146 | C9H8NO | C8H7N1 |
| Lys | 13.17 | 276 | 244,212,142,88 | 142 | C7H12NO2 | C5H8N1 |
| His | 6.01 | 285 | 254,226,210,194,140,95,81 | 210 | C8H6N2O5 | C6H4N2O |
| Tyr | 6.04 | 267 | 252,236,220,192,165,146,121 | 220 | C11H10NO4 | C8H6NO |
| Trp | 7.00 | 276 | 130 | 130 | C9H8N | C9H8N |

(a) Arginine was not detected following derivatization by this method, and threonine and serine were detected as N-MOC methyl esters with methyl hydroxylether side chains (Thr-OMe and Ser-OMe), (b) major ion used for detection of the specific amino acid, (c) chemical formula of detected fragment ions used for MIDA calculation, (d) elements in the detected fragment ions that are originally from amino acids.
