# Supplementary material for: An automated growth enclosure for metabolic labeling of Arabidopsis thaliana with 13C-carbon dioxide - an in vivo labeling system for proteomics and metabolomics research
Source: Proteome Sci. 2011 Feb 10;9:9. doi: 10.1186/1477-5956-9-9 (PMC3046907; doi:10.1186/1477-5956-9-9)
Supplement: Additional file 4 — User's manual for the enclosure system. Detailed user's manual describes set-up and operation of the enclosure. Several photographs are included at the end of the manual for reference. [file 1477-5956-9-9-S4.DOCX]

**^13^CO_2_ plant labeling system**

**User’s manual**

**Version 1.3**

**January 22, 2011**

**Protein Turnover Group**

**Department of Horticultural Science**

**Department of Plant Biology**

**University of Minnesota**

***Supported by the U.S. National Science Foundation,***

***Plant Genome Program, grant DBI 0606666***

**CONTENT**

1. **Overview**
2. **System design**
   1. **Hardware**
   2. **Control system**
   3. **Power system**
3. **Operation of the system**
   1. **Power on**
   2. **Open control VI**
   3. **Enclosure purge**
   4. **Replacement of ethylene scrubber**
   5. **Adding ^13^CO_2_**
   6. **Cold plate temperature setup**
4. **Preparation for plant growth**
   1. **Preparation of acid-washed sand**
   2. **Preparation of rockwool blocks**
   3. **Preparation of hydroponic medium**
   4. **Stratification of seeds**
   5. **Sowing seeds**
5. **Plant labeling with ^13^CO_2_**
   1. **Plant tray setup**
   2. **Lighting**
   3. **Enclosure sealing**
   4. **Air purge**
6. **Troubleshooting**
7. **Supplemental pictures**

***1. Overview***

This close plant growth chamber is designed and built for plant labeling studies with ^13^CO_2_. Plants can be fully labeled starting from seed in the chamber then chased with ambient CO_2_ of natural abundance or pulse-labeled with ^13^CO_2_ after growing with CO_2_ of natural abundance.


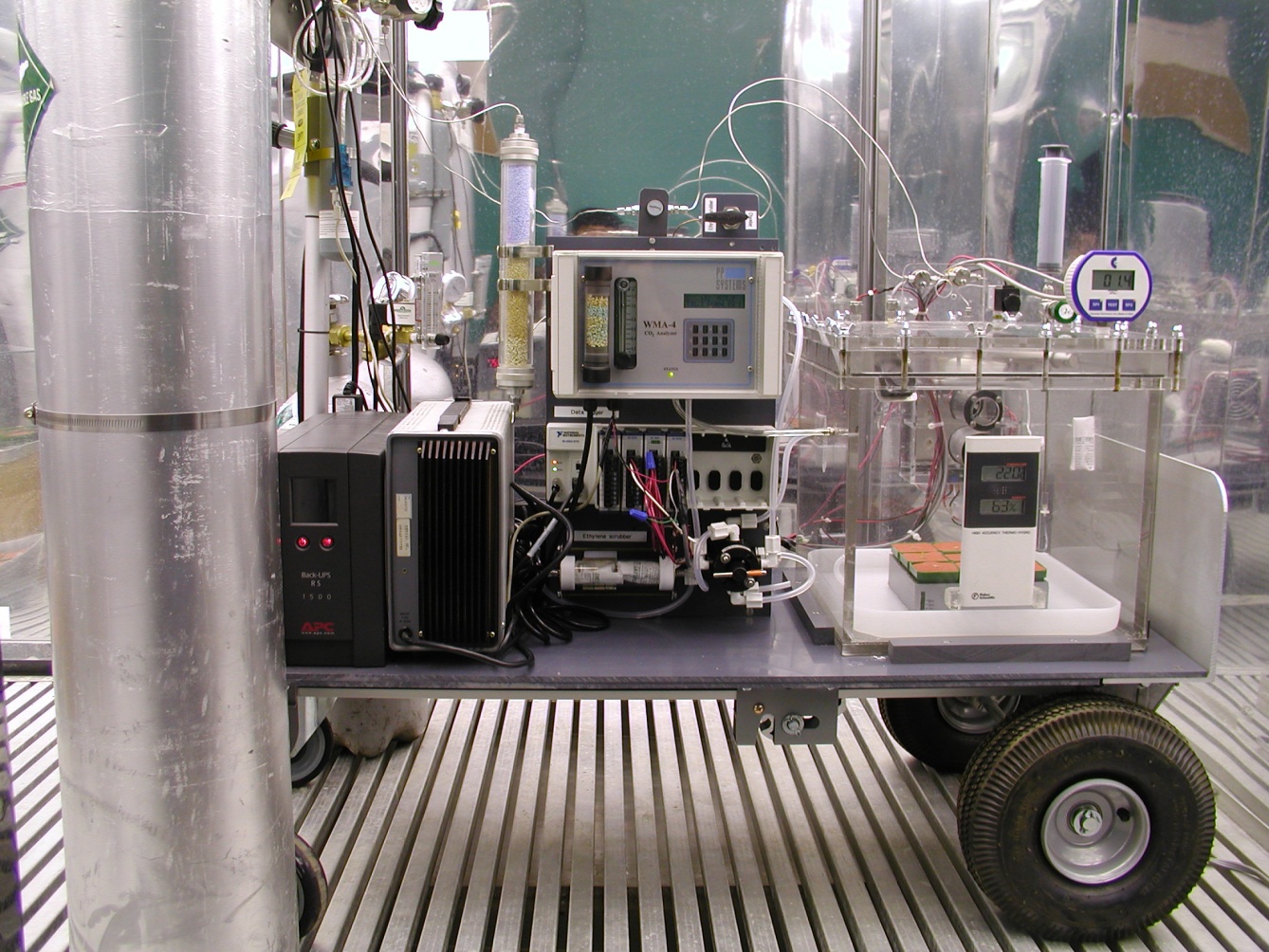


Figure 1. A front view of the ^13^CO_2_ labeling system.

***2. System design***

***2.1. Hardware setup***

This closed growth chamber was built with thick plexiglass and has a two-floor design. A small plexiglass chamber with a cold plate installed on one side of its walls is connected to the main chamber by one inlet plexiglass pipe and one outlet aluminum pipe. This cold box is designed to control the humidity of the main chamber by removing air from the main chamber using a fan onto the cold plate such that the excess moisture in the air can be condensed on the surface of the cold plate. Two solenoid valves located on the lid of the chamber control CO_2_-free air and CO_2_ gas flows. In addition, a pressure relief valve, pressure sensor and humidity/temperature sensor are installed on the lid. The CO_2_ analyzer manufactured by PP Systems is located outside the chamber and a pneumatic diaphragm pump inside the analyzer continuously circulates air from the growth chamber thru a small air chamber where the CO_2_ sensor is located. After sampling, the air flows back to the growth chamber. An ethylene scrubber is installed inline after CO_2_ sensor and the air coming out from the CO_2_ sensor is directed by a stack 4-way valve to either the ethylene scrubber or to a bypass. This valve design allows for easy replacement of the ethylene scrubber bags in the column while an experiment is running. After replacement, the scrubber column together with the fresh bags containing potassium permanganate pellets are purged by CO_2_ free air that is directed by a manually operating 3-way valve located at the top of the CO_2_ sensor. The CO_2_ free air purchased from Minneapolis Oxygen Company contains less than 1ppm CO_2_ but is further polished by a CO_2_ scrubber column before use to supply the chamber or traps for purging or maintaining the slightly positive pressure of the chamber. To prevent CO_2_ gas levels from overshooting, the flow rate of the CO_2_ gas is controlled by a needle valve set at the lowest rate. The outlet pressure of ^13^CO_2_ was controlled by the regulator hooked up directly on the lecture bottle. A pressure of around 10 psi was found to be ideal to finely control the flow rate of ^13^CO_2_. The flow rate of CO_2_ free air is not valve controlled but the outlet pressure is set between 100 and 120 psi and the flow is somewhat restricted by the narrow bore stainless steel gas line.

***2.2. Power setup***

A12V DC power supply is used to power the thermoelectric cooler (cold plate), 2 fans inside the chamber for air circulation, PWM controller. A 24V DC power supply is used to power a 3-way solenoid valve for directing either CO_2_-free-air or CO_2_ to the chamber, a 2-way solenoid as a pressure relief valve, temperature/relative humidity sensor and chamber pressure sensor. The CO_2_ analyzer is powered by 120V AC. The power supplies, the laptop controller, the data logger as well as the CO_2_ analyzer are directly plugged into a backup power system (APC) that is plugged into the wall outlet.

***2.3. System control setup***

This system can simultaneously monitor humidity/temperature, chamber pressure and CO_2_ concentration and controlling humidity, chamber pressure and CO_2_ level by a laptop computer/controller running Labview 8.5. All signals (in current) from the sensors are acquired by a data logger, Compact DAQ, from National Instruments. The signal values are acquired and compared to set points and actions are triggered to adjust the values to the set points.

*2.3.1 The control of relative humidity in the chamber*

The humidity is controlled as described earlier by directing air onto the cold plate. When the chamber humidity is higher than the set point, a relay that controls a fan inside the cold box will turn on. The air in the main chamber where plants grow will be directed into the cold box and excess moisture can be condensed on the surface of the cold plate. The condensed water will accumulate in the box to a certain point then flow back by gravity to the tray in which the plants grow.


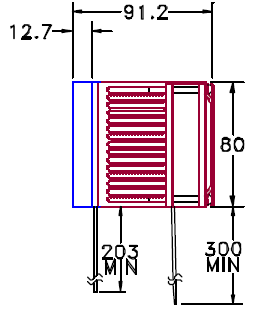


Fan

Fan

Heat sink

Bellow

All dimensions are in millimeter

Aluminum plate

Gasket

Drained to chamber

Insulation foam

Cold side

100mmx120mm surface area for this aluminum wall

Cold plate dimension: 101.6mmx80mm

Aluminum pipe

*2.3.2 The control of chamber pressure*

The chamber pressure is maintained slightly positive all the time. It is regulated by a control loop that compares actual pressure with the set minimum pressure and energizes the solenoid to inject enough CO_2_-free air into the system to maintain the set pressure. The chamber pressure is also controlled below a maximum value by a solenoid serving as a pressure relief valve. It would be energized when the chamber pressure goes over the set maximum value. The chamber pressure is kept at > 2 kPa to prevent ambient CO_2_ from entering the chamber. Chamber pressures over 5 kPa are not recommended.

*2.3.3 The control of CO_2_ levels*

The air from the chamber is continuously pumped through the CO_2_ analyzer and is monitored for CO_2_ concentration in ppm. The signal transmitted from the CO_2_ analyzer can be in either voltage or current. It has been shown that current signals are more stable than the voltage signals under most circumstances especially when surrounded by many electrical devices, thus current is used. The CO_2_ levels (SP in ppm) can be easily set at the control panel. Usually CO_2_ at 400-600 ppm for growing *Arabidopsis* gives good results. When ^13^CO_2_ is applied, the set points should be around 1/3 of the attempted concentrations because of the CO_2_ sensor used can reflect only around 1/3 of the actual CO_2_ concentrations due to the difference in the near infrared absorption of ^13^CO_2_ relative to ^12^CO_2_ for which the commercial sensor is calibrated. A PID (proportional–integral–derivative) control loop is used to control the solenoid valve for CO_2_ gas in a proportional manner. The voltage signal generated by a voltage signal module on the CompactDAQ is transmitted to a pulse-width modulator where the signal is translated into power to the load. In addition, tuning parameters were also programmed into the control loop to deal with the delay report of CO_2_ level in the chamber due to the distance between injection site and CO_2_ sensor.


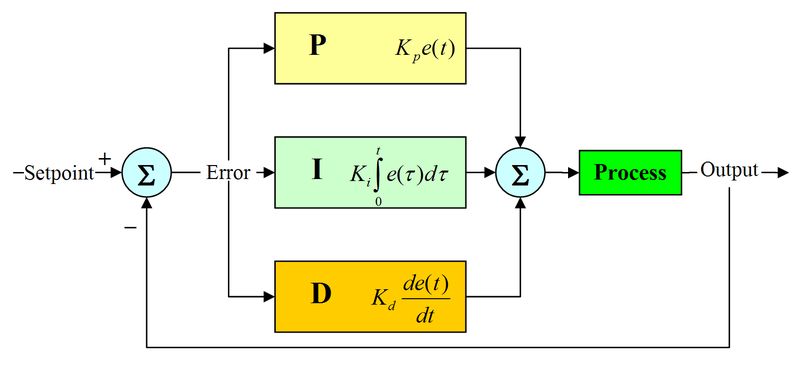


[*http://en.wikipedia.org/wiki/PID_controller*](http://en.wikipedia.org/wiki/PID_controller)

*2.3.4 The control of light intensity*

No lighting system is integrated in the system because the system was designed to be running inside a walk-in growth chamber where the lighting can be set by the control panel of the growth chamber. Even though plexiglass filters out most of the UV light as well as visible light with short wavelengths such as purple, it does not absorb visible light that is necessary for plant growth (see Figure 2).


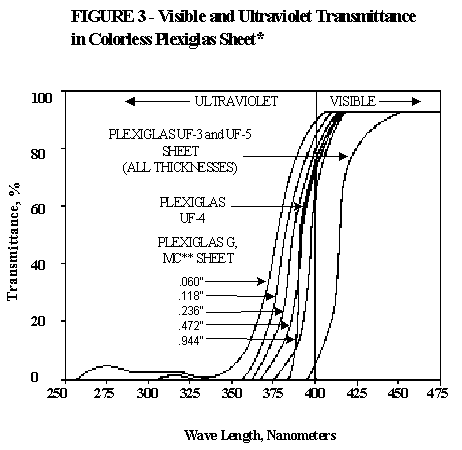


Figure 2. Visible and Ultraviolet Light Transmission in Colorless plexiglass. (from <http://www.rplastics.com/plexiglass-transmittance.html>)

The light intensity however might needs to be checked before an experiment is about to be performed. A light intensity of approximately 100 mmole m^-2^ s^-1^ is optimal to support healthy and normal-looking *Arabidopsis* plants growing in the system.


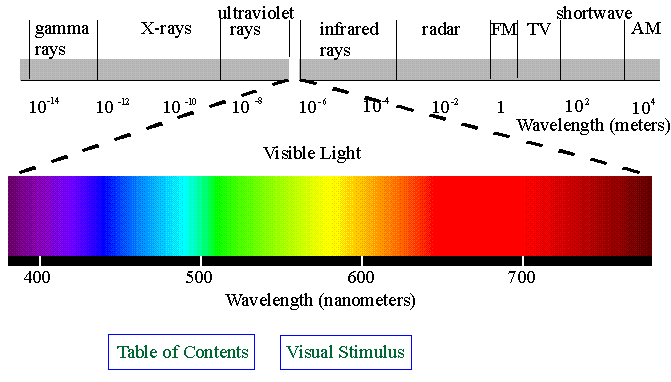


http://www.yorku.ca/eye/spectrum.gif

**3. Operation of the system**

3.1. Power on

1. Once the main power cord is connected to the electrical outlet, both the 12V and 24V power supplies that power most of the sensors, fans and cold plates, should be on. The CO_2_ analyzer, data logger and laptop controller are powered by 120V AC and are plugged directly to the power backup system. A switch in the back of the control panel where the CO_2_ sensor is mounted is used to turn on/off the two circulation fans inside the chamber. Make sure the fans are initially switched to ON.
2. Turn on the gas regulators for CO_2_-free air and CO_2_ and check the seal of gas lines using soapy water.

3.2. Open control VI (virtual instrument)

1. Turn on the labtop controller. Once Windows has booted up, double click on a LabView VI icon, called ^13^CO_2_ PID control.
2. Enter desirable parameters for relative humidity (65%), maximum (3 kPa) and minimum (2 kPa) chamber pressures, CO_2_ level (SP: 200 ppm for ^13^CO_2_ or 400ppm for natural abundance), PID tuning factor (4.99) and response delay (RPD) factor (10 ppm).
3. Press the empty arrow at the top of the front panel. The LabView VI program should then be running.

3.3. Enclosure purge with CO_2_-free air

1. Set the maximum chamber pressure at 1 kPa; at this setting the pressure relief valve will be activated and opened when pressure is higher than 1kPa and the allow air in the chamber to be purged out.
2. Press the Air Purge ON button. The solenoid for controlling air flow into the chamber will be activated.
3. Wait at least 1 hour until the CO_2_ level in the chamber goes lower than 5 ppm. In practice it is very difficult to get the CO_2_ level lowered to closer to 0 ppm, due partly to the accuracy of the CO_2_ analyzer at the lower levels.

3.4. Adding ^13^CO_2_ gas to the chamber

1. Slightly open the ^13^CO_2_ tank valve. Little or no bottle pressure shown in the regulator primary gauge is OK.
2. Adjust the outlet pressure gauge to 10 psi.
3. Open the outlet valve.
4. Adjust the needle valve that is used to control the CO_2_ flow to slightly greater than 0.
   1. Replacement of ethylene scrubber
5. Turn the stacked 4-way valve to bypass mode.
6. Disassemble the ethylene scrubber column.
7. Remove the used the sachets and put two fresh ones back.
8. Reassemble the column.
9. Switch the CO_2_ free air valve to ethylene scrubber mode and purge the ethylene scrubber with the air for at least one minute.
10. Switch the 4-way valve back to RUN mode
11. Switch the air valve back to the Chamber mode.

CO2 sensor

Ethylene scrubber

Chamber

Stack 4-way valve

Run mode

Bypass mode

Purge inlet

CO2 free air

Purge outlet

Purge inlet

CO2 free air

Purge outlet

**4. Preparation for plant growth**

*4.1. Preparation of modified Gibeaut’s solution (http://www.ag.unr.edu/Cramer/hydroponic.html)*

The macronutrient stock solutions are kept in separate containers. The iron stock solution is kept in aluminum-foil-covered or a dark-brown bottle to prevent light degradation. The 1000X micronutrient stock solution is a mixture of all micronutrients combined together in one container. Be sure to make with deionized-glass distilled water. After making the complete modified solution, the pH should be around pH 6.0 without titration. The medium then heated to 60˚C and is purged with helium gas for at least 15 min in a bottle as shown in the figure below.

| **Gib Macronutrients** | **FW** | **[STOCK]** | **STOCK**  **(g/L)** | **mL (solution)/**  **L (water)** |
| --- | --- | --- | --- | --- |
| Ca(NO_3_)_2_ x 4H_2_O | 236.15 | 1M | 236.15 | 1.50 |
| KNO_3_ | 101.11 | 1M | 101.11 | 1.25 |
| Mg(SO_4_) x 7H_2_O | 246.48 | 1M | 246.48 | 0.75 |
| KPO4 buffer, pH5.6 | 136.09 | 1M | 136.09 | 0.50 |
| Na_2_O_3_Si x 9H_2_O | 284.20 | 0.1M | 28.42 | 1.00 |
| FeCl_3_ (or Fe-EDTA) | 162.2 | 0.02 M | 3.25 | 4 |
| **Gib Micronutrients** |  |  |  |  |
| KCl | 74.56 | 50mM | 3.728 | 1.00 |
| MnSO_4_ x H_2_O | 169.01 | 10mM | 1.690 |  |
| CuSO_4_ x 5H_2_O | 249.68 | 1.5mM | 0.375 |  |
| ZnSO_4_ x 7H_2_O | 287.54 | 2mM | 0.575 |  |
| H_3_BO_3_ | 61.83 | 50mM | 3.092 |  |
| CoCl_2_ x 6 H_2_O | 237.93 | 0.01mM | 0.00238 |  |
| Na_2_MoO_4_-2H_2_O | 241.95 | 0.2mM | 0.048 |  |

*4.2. Preparation of acid-washed sand (not preferred due to poor plant growth)*

1. Industrial silica sand (Badger Mining Corporation, Berlin, WI) was used. Sand, abound 2.5 L in volume in a 5 L beaker was first washed with distilled water several times to remove water soluble contaminants. A long glass pipette with the tip cut can be prepared for this purpose. Simply connect it to a distilled water faucet by plastic tubing.
2. Decant the excess water leaving the water level at 2.5 L
3. Slowly add 300 ml of concentrated HCl to the beaker
4. Mix the acid solution by digging into the sand with the long pipette.
5. Cover the beaker with aluminum foil and let it stand for at least one day.
6. Pour out the acidic water in a sink with the tap water is running
7. Rinse the sand with distilled water by injecting water into the bottom of beaker using the long glass pipette. Move the pipette to different corners of the beaker.
8. Once the water has filled up, pour out the water and repeat step 7.
9. Repeat step 7-8 at least 10 times then let the sand sit overnight with the water.
10. Pour out the excess water and repeat step 7-8 three to four more times
11. Check the pH of the water after letting it sit for another hour.
12. It would be a safe precaution to rinse the sand again just before it is used for growing plants.

*4.3. Preparation of rockwool (preferred)*

1. Rockwool (Grodan) can be used as a preferred substitute for sand.
2. Rinse rockwool blocks with distilled water 3 times before use.

*4.4. Preparation of plant pots*

1. 2’ x 2’ square plastic pots are used. Soak the pots with ZeroTol sterilizing agent (BioSafe Systems LLC, Brentwood, TN) for a day before use.
2. Place acid-washed sand or rockwool block in the pots.
3. Rinse with distilled water at least 2 times.
4. Place the pots on a holding tray and add 10 ml of 0.5X Gib medium onto the top of the sand or rockwool.
5. Layer a cover made of silicon rubber as shown in the figure below.

*4.5. Stratification and sowing of Arabidopsis seeds*

1. *Arabidopsis* seeds after being rinsed with distilled water are left in a cold room (4 °C) for at least one day before sowing on sand or rockwool blocks in small pots on a solution holding tray.
2. Layer a silicon rubber cover with a hole in the center is placed on the top of the sand or rockwook block
3. Next, add a small amount of wet sand to the center of the holes, but don’t fill the holes up fully with the sand. Alternatively, you may skip this step and sow seeds directly onto the sand or rockwool.
4. Take one seed at a time using a pipette and drop the seeds into the holes individually

*5.0. Preparation of helium-purged medium and medium injection to the chamber*

1. Set up a glass bottle with two valves as shown in the figure below.
2. Pour 300 ml of Gib medium into the bottle and seal with the cap.
3. Open the two valves and connect helium tubing to the inlet valve
4. Place the medium on a hot plate and bring to 60˚C with stir bar mixing for 30 min while purging with helium.
5. Close two valves and cool medium using tap water or let it sit at room temperature until it has cooled.
6. When withdrawing the medium, connect a CO_2_ scrubber, as shown in figure, to the outlet valve and a 60 ml syringe to the inlet valve, then pull the syringe piston to fill.
7. Inject the medium into the chamber through the designated valve.

**5. Plant labeling with ^13^CO_2_**

1. Open the chamber
2. Disconnect the tray from the water-recycle tubing of the dehumidifier box and clean it up with detergent then rinse with water
3. Prepare the pots and seeds as described in 4.4 and 4.5
4. Connect the tray back to the water-recycle tubing
5. Check the light intensity with a light meter before the chamber is sealed.
6. Make sure the ethylene scrubber is fresh. Replace it when necessary
7. Seal the chamber
8. Inject helium purged *Gibeaut’s* medium to the tray from the feeding valve. See medium preparation for details.
9. Set the humidity at 50% to activate the dehumidifier fan in order to circulate the air in the dehumidifier box to the main chamber when purging
10. Purge the chamber with CO_2_ free air until the CO_2_ reading is below 3 ppm
11. Set the CO_2_ concentration at 100 ppm (the actual CO_2_ concentration for ^13^CO_2_ would be approximately 300 ppm).
12. Slowly turn on the ^13^CO_2_ gas regulator until the pressure is about 10 psi
13. Monitor the CO_2_ level approaching 100 ppm on the controller. If it increases too fast, adjust the valve to lower the flow rate.
14. Wait until the CO_2_ level reading is stable at 100 ppm.
15. Stand for at least 1 hour.
16. Repeat steps 8-14 twice.
17. Set the maximum and minimum chamber pressure at 3 kPa and 2 kPa, respectively and the CO_2_ concentration at 200 ppm (the actual CO_2_ concentration for ^13^CO_2_ would be approximately 600 ppm).
18. Monitor the cold plate temperature occasionally to make sure the dehumidifier is working properly.
19. Make sure the light intensity is adequate so that the plants would have optimal growth.

**6. Troubleshooting**

*Q1. Seedlings stop growing or flowers are aborted.*

A1. The capability of ethylene scrubber may have been exceeded. Change the ethylene scrubber sachets as required to avoid this problem.

*Q2. Cannot bring the chamber humidity down to the set point.*

A2. Check the temperature of the cold plate on the cold box. Re-adjust the temperature of the cold temperature to at least below 15°C. Refer to the Operation Manual for the Model TC-48-20 Thermoelectric Cooler Temperature Controller for specific details.

*Q3. CO_2_ gas level is overshooting.*

A3. Try to turn down the outlet pressure of the CO_2_ gas regulator to less than 10 psi and adjust the CO_2_ flow rate on the scale as low as possible.

*Q4. The labeling ratios of plant metabolites and proteins are low.*
A4. (1) Make sure to purge the CO_2_ line with ^13^CO_2_ at least twice before you start to grow plants in the chamber. (2) Remember to use FeCl_3_ or Fe-EDTA as iron sources of the medium but never Fe-citrate. (3) Purge the medium with helium gas well before each use. (4) Change the CO_2_ scrubber medium more often (5) Check for chamber or connection leaks.

**Supplements**

1. **Cartoon of the chamber system**


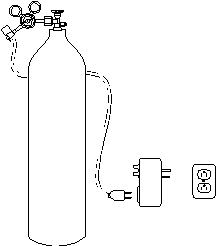

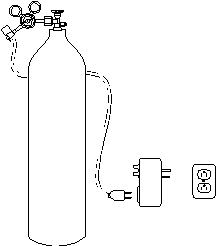


Tray

^13^CO_2_

Air

O_2_/N_2_

CO_2_ sensor

solenoid

Laptop controller w/ PID

Pressure sensor

Hoagland Solution

Fan

condensed water

Electronic

Cooling unit

Plugs

Defrost

timer

Thermometer

Heat sink

For chamber purge

Air pressure control valve

Filter

CO2 scrubber


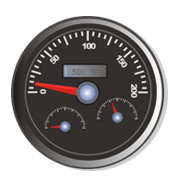


RH/Temp sensor

Ethylene scrubber

Data logger

System on a cart

Cold plate


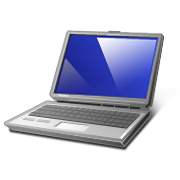


Laptop controller

Air

DAQ

Backup power

Chamberrrr

CO2 sensor

12V power supply

Backup power supply


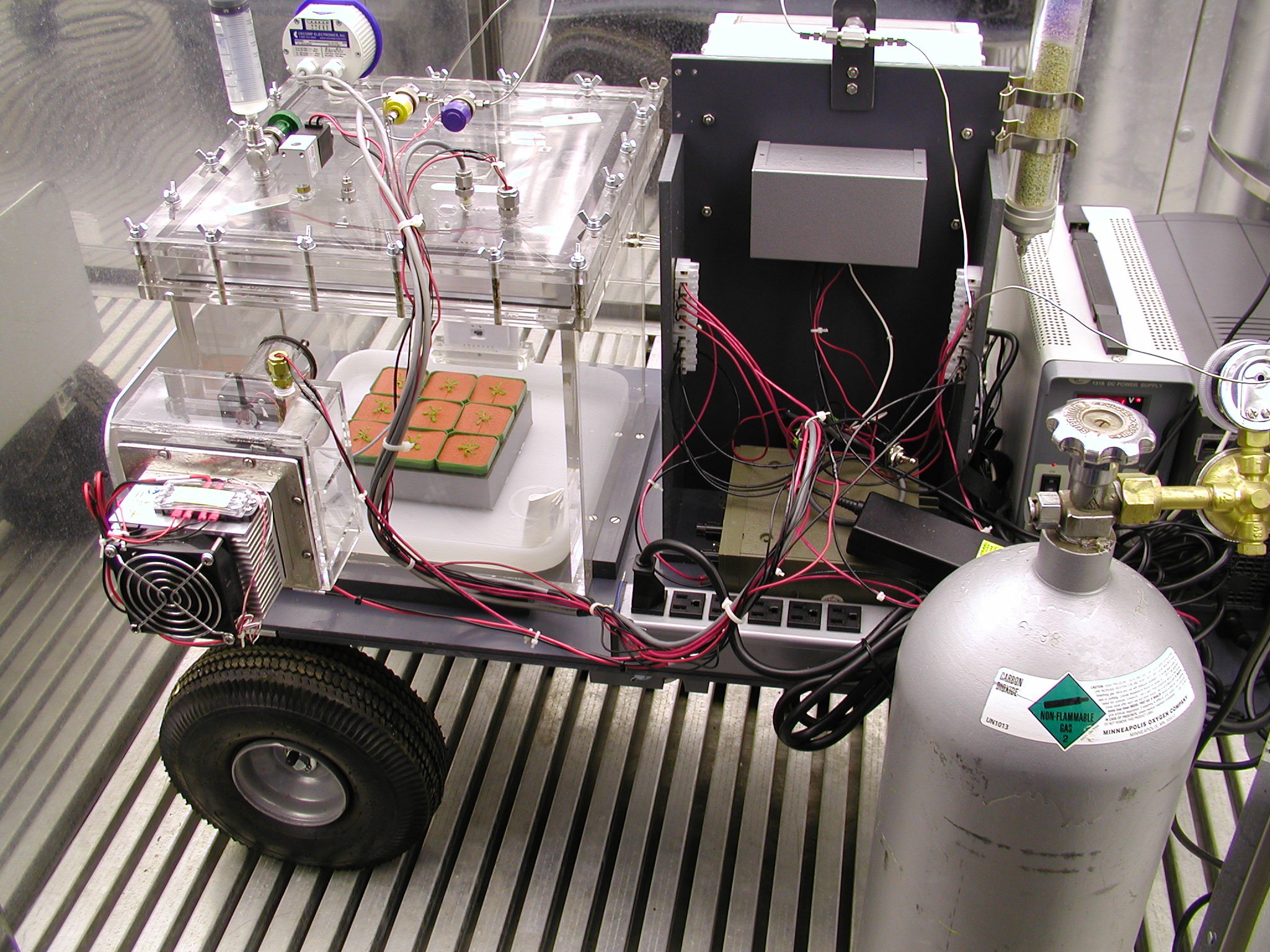


PWM

controller

^12^CO_2_ tank

12V power

supply

24V power

supply

Cold plate

dehumidifier

Temperature controller

(underneath the box)


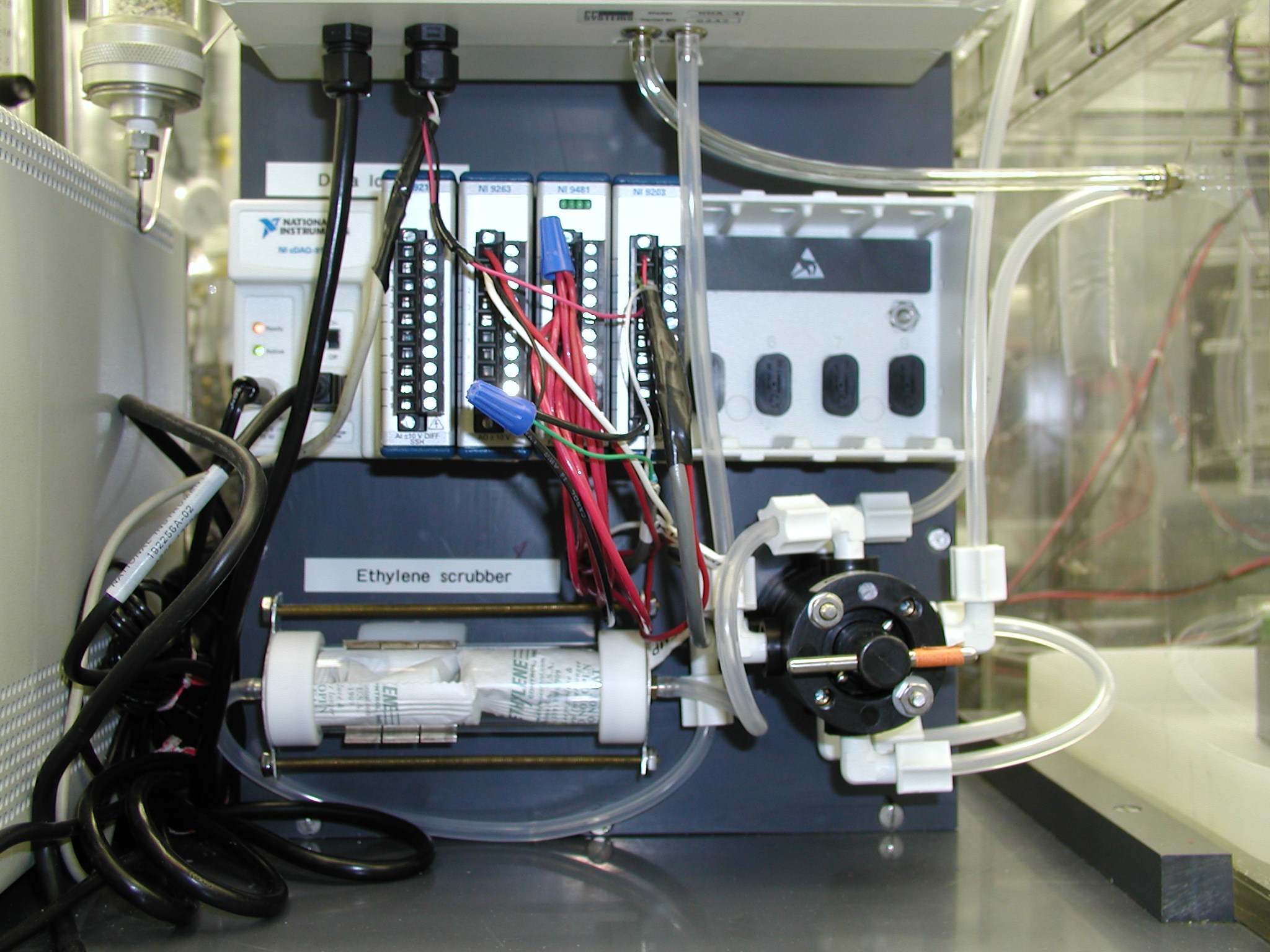


Compact DAQ

Ethylene scrubber

Stacked 4-way valve

Chamber

Air out

Air in

12V power supply

CO2 analyzer


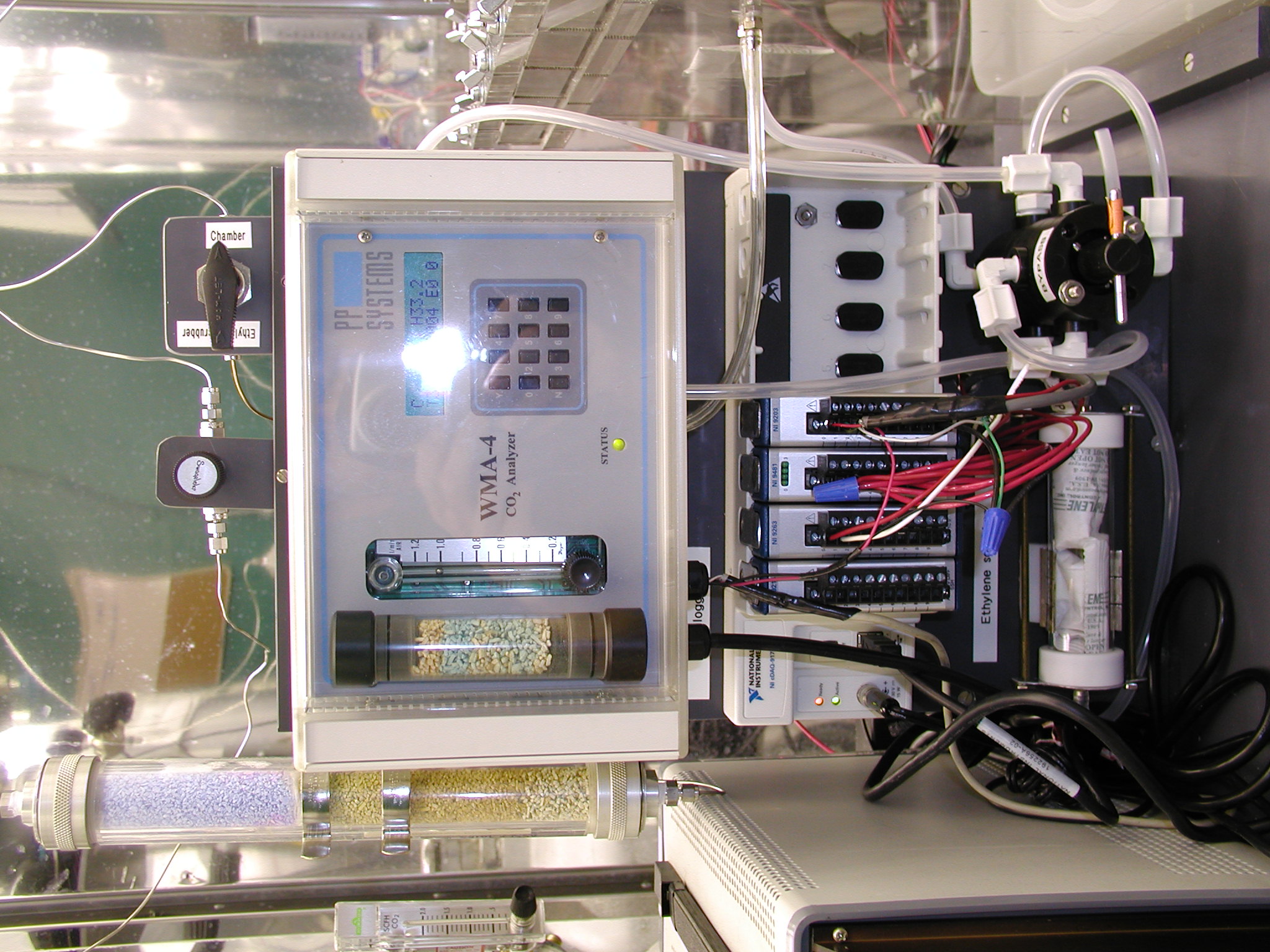


CO_2_ flow control valve

CO_2_ free air directing valve

CO_2_ analyzer

CO_2_ scrubber for

CO_2_ free air

CO_2_ scrubber for

autozeroing of CO_2_ analyzer


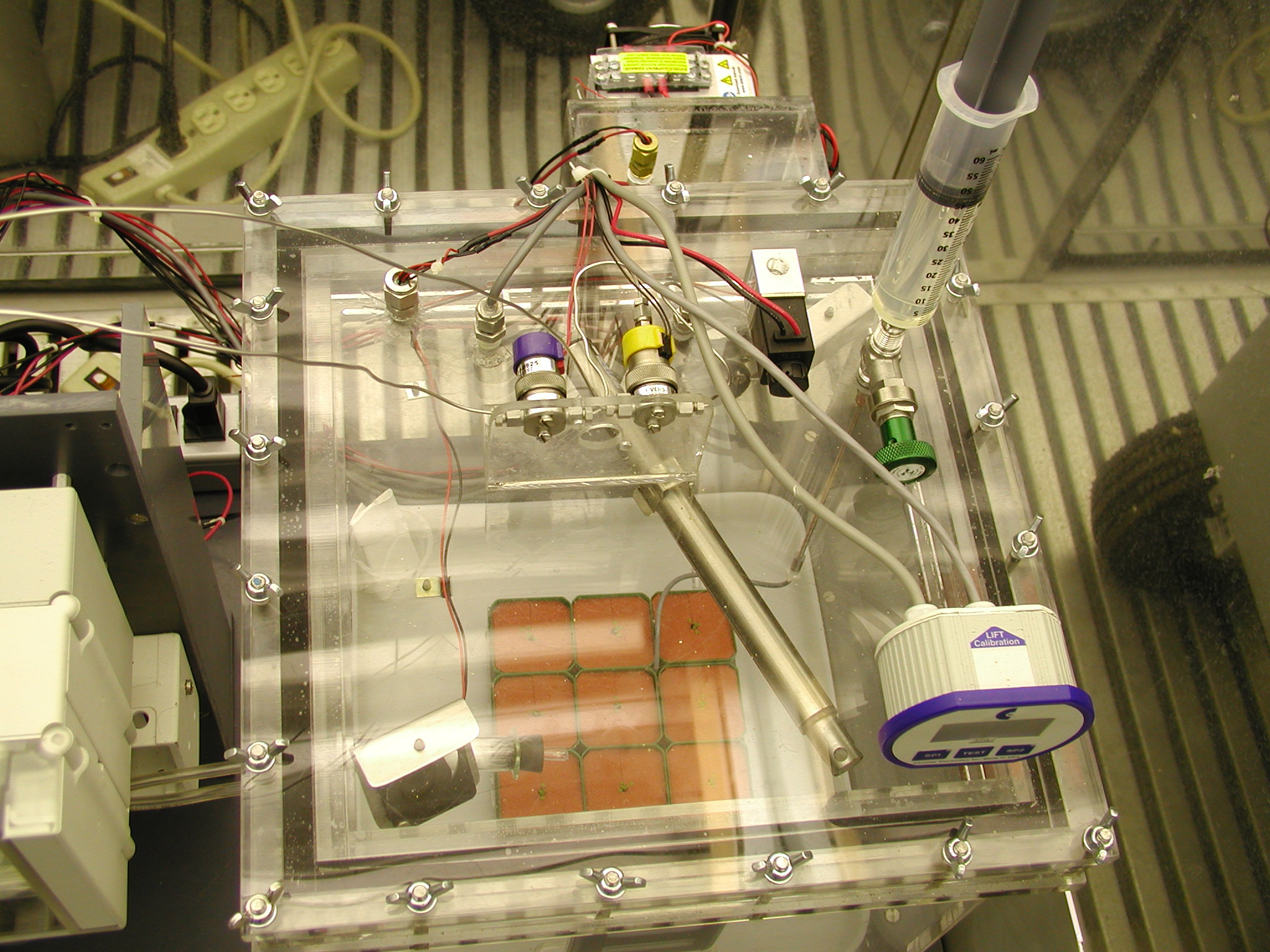


Medium syringe syringe

CO_2_ analyzer

Humidity/Temp sensor

Circulation fan

2-way valve as

Pressure relief valve

3-way valve for

gas directing

2-way valve for

CO_2_ control

Pressure sensor


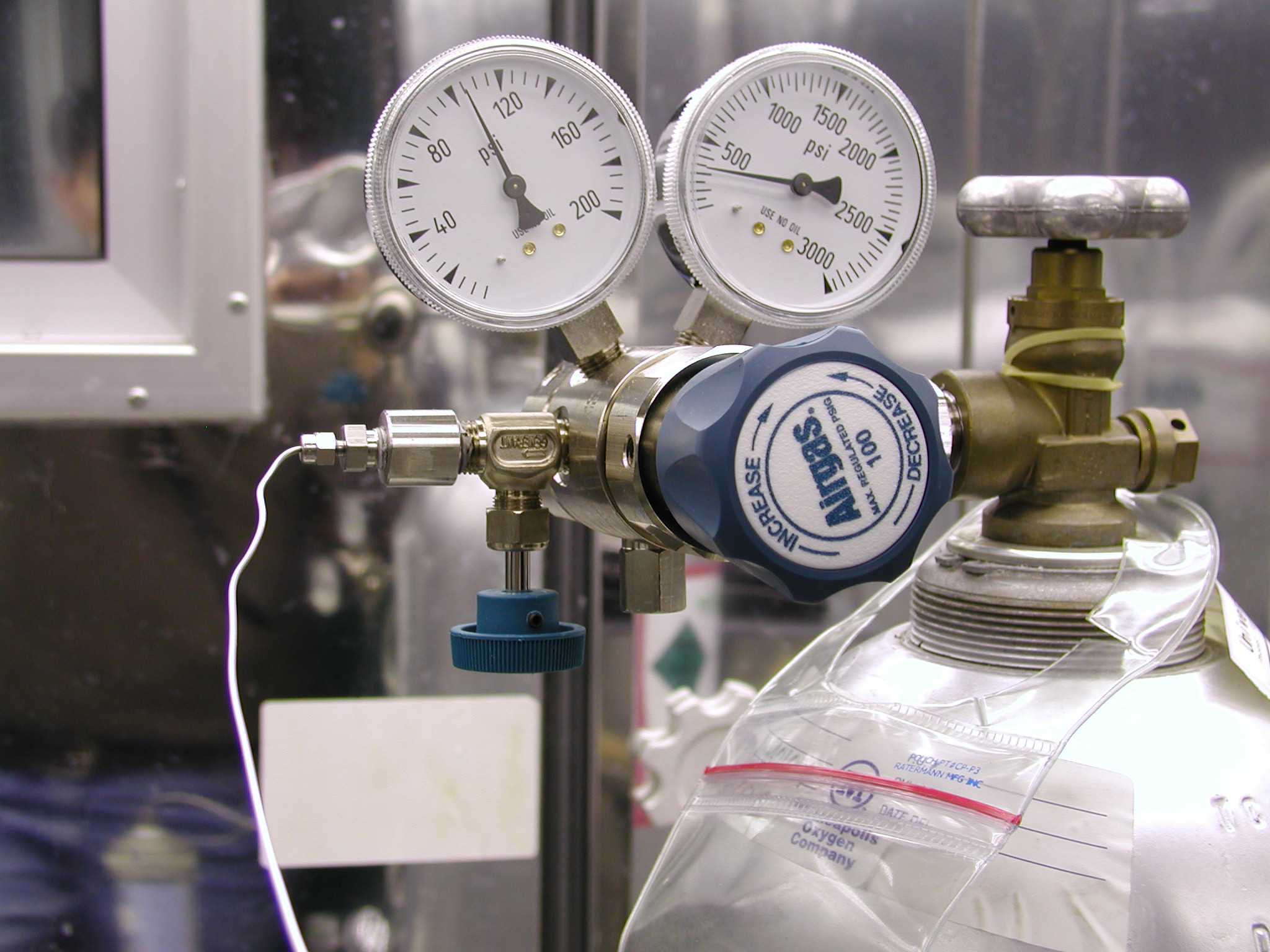

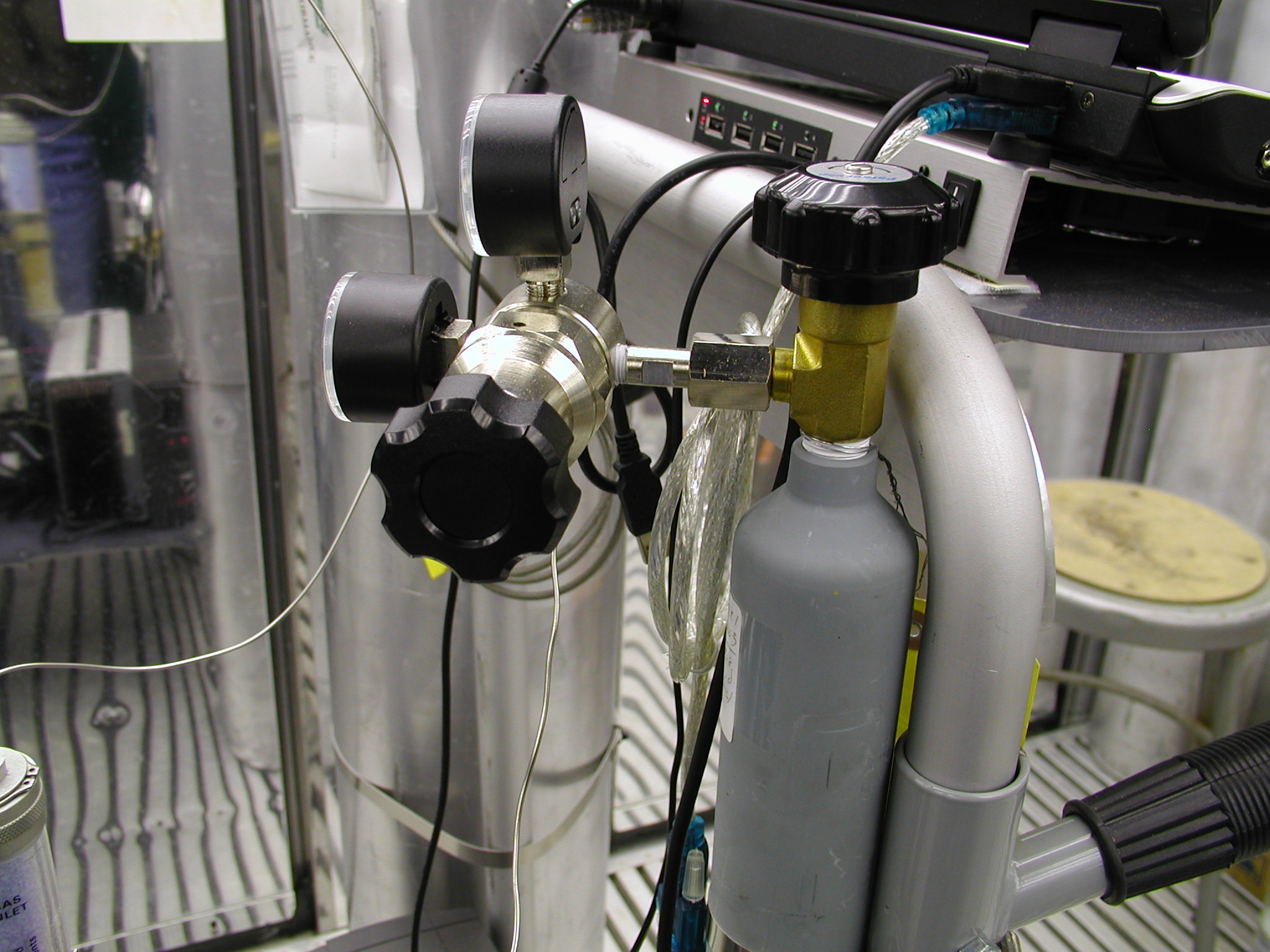


Gas regulator setup for CO_2_ free air.

Output pressure: 100 psi

Gas regulator setup for ^13^CO_2_ lecture bottle. Output pressure: 10 psi


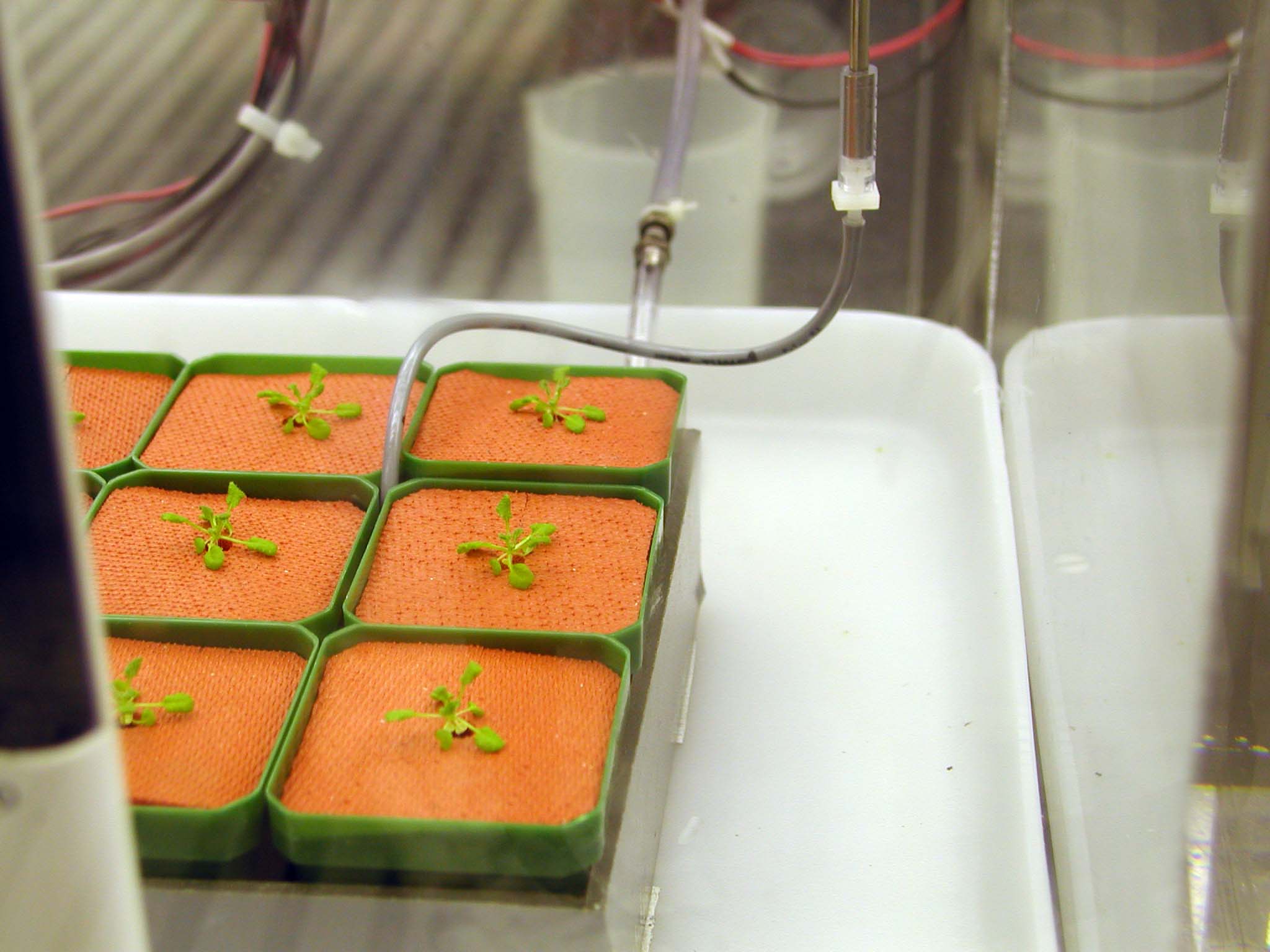


Water recycle tubing

Medium feeding tubing

Silicone rubber cover


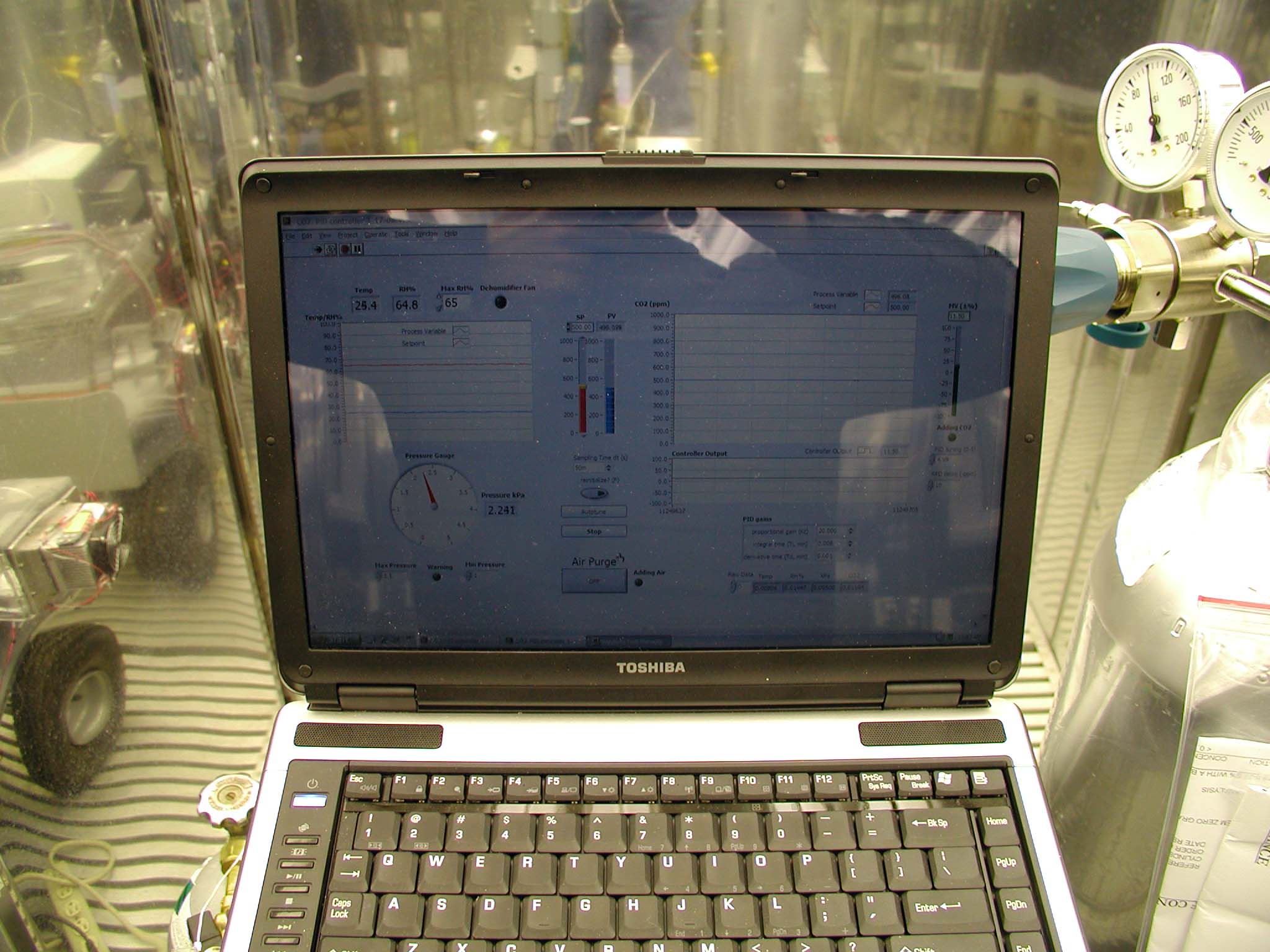


Chamber control VI in Labview 8.5

Laptop controller

Gas regulator

**
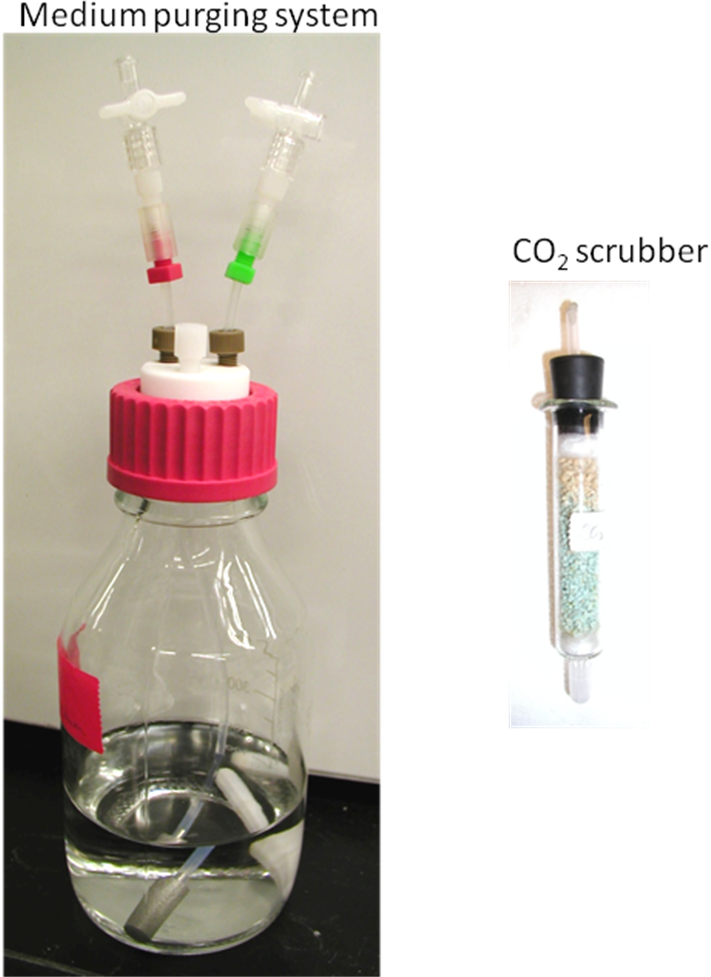
**

**Seedlings grown in the enclosure for 3 weeks from seed**

**
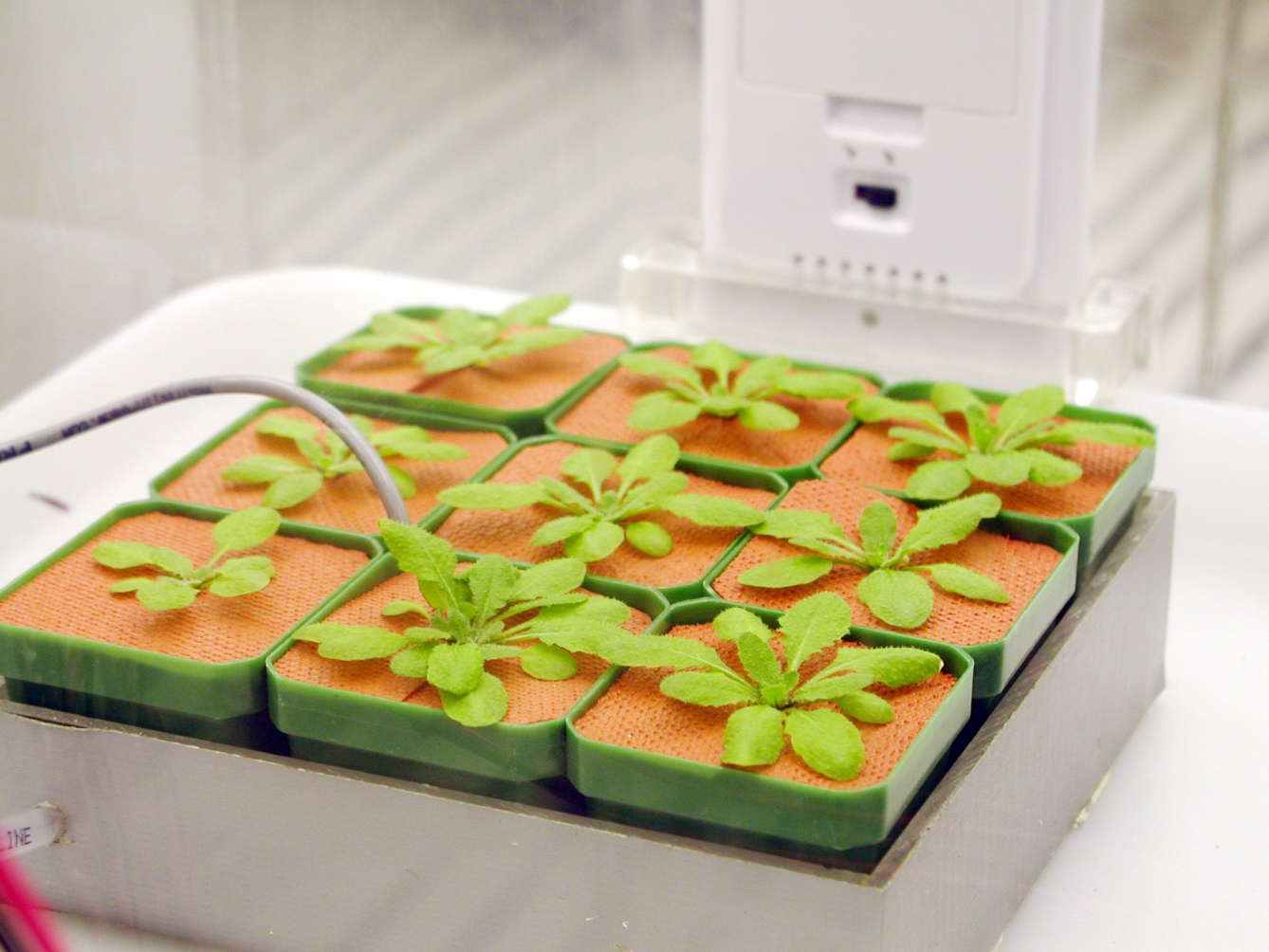
**
